# Sodium salt medium-chain fatty acids and *Bacillus*-based probiotic strategies to improve growth and intestinal health of gilthead sea bream (*Sparus aurata*)

**DOI:** 10.7717/peerj.4001

**Published:** 2017-12-04

**Authors:** Paula Simó-Mirabet, M. Carla Piazzon, Josep Alvar Calduch-Giner, Álvaro Ortiz, Mónica Puyalto, Ariadna Sitjà-Bobadilla, Jaume Pérez-Sánchez

**Affiliations:** 1 Nutrigenomics and Fish Growth Endocrinology Group, Institute of Aquaculture Torre de la Sal, CSIC, Castellón, Spain; 2 Fish Pathology Group, Institute of Aquaculture Torre de la Sal, CSIC, Castellón, Spain; 3 Evonik Nutrition and Care GmbH, Hanau-Wolfgang, Germany; 4 NOREL S.A., Madrid, Spain

**Keywords:** Medium-chain fatty acid, Teleost, Probiotic, Intestinal health, *Bacillus amyloliquefaciens*, DICOSAN

## Abstract

**Background:**

The increased demand for fish protein has led to the intensification of aquaculture practices which are hampered by nutritional and health factors affecting growth performance. To solve these problems, antibiotics have been used for many years in the prevention, control and treatment against disease as well as growth promoters to improve animal performance. Nowadays, the use of antibiotics in the European Union and other countries has been completely or partially banned as a result of the existence of antibiotic cross-resistance. Therefore, a number of alternatives, including enzymes, prebiotics, probiotics, phytonutrients and organic acids used alone or in combination have been proposed for the improvement of immunological state, growth performance and production in livestock animals. The aim of the present study was to evaluate two commercially available feed additives, one based on medium-chain fatty acids (MCFAs) from coconut oil and another with a *Bacillus*-based probiotic, in gilthead sea bream (GSB, *Sparus aurata*), a marine farmed fish of high value in the Mediterranean aquaculture*.*

**Methods:**

The potential benefits of adding two commercial feed additives on fish growth performance and intestinal health were assessed in a 100-days feeding trial. The experimental diets (D2 and D3) were prepared by supplementing a basal diet (D1) with MCFAs in the form of a sodium salt of coconut fatty acid distillate (DICOSAN^®^; Norel, Madrid, Spain), rich on C-12, added at 0.3% (D2) or with the probiotic *Bacillus amyloliquefaciens* CECT 5940, added at 0.1% (D3). The study integrated data on growth performance, blood biochemistry, histology and intestinal gene expression patterns of selected markers of intestinal function and architecture.

**Results:**

MCFAs in the form of a coconut oil increased feed intake, growth rates and the surface of nutrient absorption, promoting the anabolic action of the somatotropic axis. The probiotic (D3) induced anti-inflammatory and anti-oxidant effects with changes in circulating cortisol, immunoglobulin M, leukocyte respiratory burst, and mucosal expression levels of cytokines, lymphocyte markers and immunoglobulin T.

**Discussion:**

MCFA supplementation showed positive effects on GSB growth and intestinal architecture acting mainly in the anterior intestine, where absorption takes place. The probiotic *B. amyloliquefaciens* CECT 5940 exhibited key effects in the regulation of the immune status inducing anti-inflammatory and anti-oxidant effects which can be potentially advantageous upon infection or exposure to other stressors. The potential effects of these feed additives in GSB are very promising to improve health and disease resistance in aquaculture.

## Introduction

Aquaculture is an expanding industry with 73.8 million tonnes produced globally in 2014 (FAO, 2016). The increased demand for fish protein has led to the intensification of aquaculture practices increasing the risk of infectious diseases (Segner et al., 2012). In this regard, the growth of the aquaculture industry is hampered by nutritional and health factors which affect growth performance. To sort out these problems, antibiotics have been used for many years in the prevention, control and treatment against disease as well as growth promoters to improve animal performance (Done, Venkatesan & Halden, 2015). However, due to the occurrence of antibiotic cross-resistance, the European Union and other countries have completely or partially banned their use for growth and disease prevention purposes (January 2006; Regulation 1831/2003/EC). Hence, a number of alternatives, including enzymes, prebiotics, probiotics, phytonutrients and organic acids used alone or in combination have been proposed for the improvement of host immunity or animal growth and production (Seal et al., 2013).

The use of organic acids as feed additives has been in progress for over four decades, and several studies have proved their antimicrobial and growth-promoting action in swine and poultry (Suiryanrayna & Ramana, 2015; Khan & Iqbal, 2016). Currently, there is also commercial and research interests in the use of organic acids in aquafeeds to improve growth performance, nutrient utilization and disease resistance in commercially important farmed fish. Many studies have reported that some short-chain fatty acids (SCFAs) and their salts or mixtures can significantly enhance growth performance and health status of fish (Ng & Koh, 2016) or be benefitial in reverting detrimental effects produced by low inclusion levels of fish meal and fish oil (Benedito-Palos et al., 2016; Estensoro et al., 2016). Another type of organic acids often used as nutritional supplements are medium-chain fatty acids (MCFAs), which are highly abundant in oil distillates from coconut oil, palm kernels and milk. MCFAs have been suggested to have a role in immunological regulation (Wang et al., 2006), antibacterial activity (Bergsson et al., 2001; Skřivanová et al., 2009) and can also improve gut development, enhancing performance in piglets (Hanczakowska et al., 2016). The physiological consequences of supplementing fish aquafeeds with medium-chain triglycerides are poorly studied. However, a recent study in GSB demonstrated the up-regulation of different immune-related genes in skin of fish feed with a palm oil-supplemented diet, which could be considered a good mechanism to enhance humoral immunity in fish skin (Cerezuela et al., 2016). In the same way, stimulation of several immune parameters has been detected in European sea bass (*Dicentrarchus labrax*) fed with a diet enriched with extracts obtained from the date palm fruits (Guardiola et al., 2016).

Another potential strategy to replace antibiotics is the use of probiotics. Although the first application of probiotics in aquaculture feeds was more than three decades ago, their use has recently regained considerable attention due to their ability to act as growth promoters and their positive effects in disease control, nutrient digestion, reproduction, stress tolerance as well as in the improvement of water quality (Zorriehzahra et al., 2016). Among probiotics, the genus *Bacillus* has been used in humans and animals due to their ability to produce antimicrobial substances and their sporulation capacity, conferring them a double advantage in terms of survival in different habitats (Abriouel et al., 2011). Specifically, the spores of *Bacillus amyloliquefaciens* have been used as probiotics in poultry feeds, because they reduce the effect of pathogenic bacteria such as *Clostridium perfringens*, *Escherichia coli* and *Yersinia* and thus decrease poultry mortality (Diaz, 2007; Commission Regulation (EC) No. 1292/2008). Among the few studies addressing the use of *B. amyloliquefaciens* in aquafeeds, improved growth performance (Ridha & Azad, 2012) and enhanced immune status and disease resistance (Selim & Reda, 2015) have been described in Nile tilapia (*Oreochromis niloticus*). Similar results have also been reported for European eel (*Anguilla anguilla*) (Lu et al., 2011), catfish (*Ictalurus punctatus*) (Ran et al., 2012) and Indian major carp (*Catla catla*) (Das et al., 2013).

The aim of the present study was to evaluate the potential benefits of two commercially available feed additives, one based on MCFAs from coconut oil and another a *Bacillus*-based probiotic, in GSB, a marine farmed fish of high value for the Mediterranean aquaculture. To pursue this issue, we assessed their effects on growth performance and intestinal health in a 14-weeks feeding trial. To our knowledge, this is the first report addressing in the same study the effects of MCFAs with great growth-promoting action, and a *Bacillus*-based probiotic with promising effects on immune maintenance and disease resistance in a farmed fish.

## Material & Methods

### Experimental diets

The basal control diet (D1) and two different experimental diets (D2, D3) were formulated and delivered by SPAROS LDA (Portugal) (Table 1). D1 diet (45% protein and 18% fat), contained fish meal, blood proteins and poultry meal as the main source of dietary proteins. Plant ingredients as a blend of soy protein, wheat gluten, corn gluten, soybean meal, rapeseed meal, wheat meal and pea starch were included at 42%. Fish oil (SAVINOR, Portugal) was added at 13.9%. The experimental diets (D2 and D3) were prepared by supplementing D1 diet with two different commercial preparations: (i) medium-chain fatty acids in the form of a sodium salt of coconut fatty acid distillate (DICOSAN^®^, Norel) rich on C-12 was added as a powder to D2 diet at 0.3%; (ii) the probiotic *Bacillus amyloliquefaciens* CECT 5940 was added “top coated” to D3 diet, being mixed with oil and sprayed at the appropriate rate to match the final dose at 0.1%. All diets were supplemented with antioxidants, a mineral-vitamin mix and DL-methionine.

**Table 1:** Experimental diet composition. Ingredients of basal/control diet (D1). Experimental diets were formulated on the same composition of D1 with 0.3% DICOSAN for diet D2 or 0.1% probiotic (*Bacillus amyloliquefaciens*) for diet D3.

Ingredient (%)	D1
Fishmeal LT Diamante	7.5
Fishmeal 60	20
CPSP 90	2.5
Porcine blood meal	5
Poultry meal 65	7
Soy protein concentrate	5
Wheat Gluten	6
Corn gluten	5
Soybean meal 48	9
Rapeseed meal	5
Wheat meal	7
Pea starch	5
Fish oil—SAVINOR	13.9
Vit & Min Premix	1
Binder (Kieselghur)	0.5
Antioxidant powder (Paramega)	0.2
Sodium propionate	0.1
DL-Methionine	0.3

### Fish, feeding trial and sampling collection

Juvenile GSB of Atlantic origin (Tina Menor, Santander, Spain) of 4–5 g initial body weight (May 2015) were acclimatized for more than two months to the indoor experimental facilities of the Institute of Aquaculture Torre de la Sal (IATS-CSIC, Spain). During this initial period, fish were fed with standard diets (Inicio Forte 824 1.9 mm; BioMar, Dueñas, Spain). Then, fish of 29.6 ± 0.32 g initial mean body weight (±SEM) were distributed in 500 l tanks in triplicate groups of 50 fish each. Each experimental diet was offered to visual satiety (one time per day) six days per week from June to August and five days per week from September to October in a 100-days feeding trial). Feed intake was recorded weekly and fish were counted and group-weighed every 4–6 weeks. Oxygen content of outlet water remained higher than 75% saturation, and day-length and water temperature followed the natural changes at IATS latitude (40°5′N; 0°10′E), varying from 24 °C in later June to 22 °C in early October with a maximum peak of 28 °C at the end of July.

At the end of the trial, overnight fasted fish (6 fish per tank, 18 per experimental condition) were randomly sampled and anaesthetized with 3-aminobenzoic acid ethyl ester (MS-222, 0.1 g/l; Sigma, St. Louis, MO, USA) for blood and tissue collection. Blood was quickly drawn from caudal vessels with heparinized syringes. One aliquot was directly used for measurements of leukocyte respiratory burst and hematocrit/hemoglobin determinations. The remaining blood was centrifuged at 3,000 ×g for 20 min at 4 °C, and plasma samples were frozen and stored at −80 °C until biochemical and immunological assays were done. Prior to tissue collection, anaesthetized fish were killed by cervical section, and liver, viscera and mesenteric fat were weighed. Intestine was taken for weight and length measurements. Representative portions of anterior and posterior intestinal segments were rapidly taken, frozen in liquid nitrogen and stored at −80 °C until RNA isolation. Additional samples of liver and intestinal segments were taken for histological processing.

All procedures were approved by the Ethics and Animal Welfare Committee of the Institute of Aquaculture Torre de la Sal according to national (Royal Decree RD53/2013) and EU legislation (2010/63/EU) on the handling of experimental animals.

### Hematological, biochemical and immunological blood analyses

Hemoglobin concentration was determined with a HemoCue B-Hemoglobin Analyser^®^ (Aktiebolaget Leo Diagnostics, Helsigborg, Sweden), which uses a modified azide methemoglobin reaction for Hb quantification. The hematocrit (Hc) was measured after centrifugation of blood in heparinized capillary tubes at 13,000 ×g for 10 min. Plasma glucose was determined by the glucose oxidase method (ThermoFisher Scientific, Waltham, MA, USA) according to manufacturer’s instructions. Plasma triglycerides (TGs) were determined using lipase/glycerol kinase/glycerol-3-phosphate oxidase reagent (ThermoFisher Scientific, Waltham, MA, USA). Total cholesterol was measured using cholesterol esterase/cholesterol dehydrogenase reagent (ThermoFisher Scientific, Waltham, MA, USA). Total plasma proteins were measured using BioRad protein reagent (Hercules, CA, USA) with bovine serum albumin as standard. Total antioxidant capacity was measured as Trolox activity using a microplate assay kit (709,001, Cayman Chemical, Ann Arbor, MI, USA).

Induction of the respiratory burst (RB) activity of blood leukocytes was measured directly from heparinized blood as previously reported (Saera-Vila et al., 2009). Briefly, 4 µl of heparinized blood were diluted with 96 µl Hank’s balanced salt solution pH 7.4 with calcium and magnesium (HBSS++; Gibco, ThermoFisher Scientific, Waltham, MA, USA) in duplicates in white 96 well plates (NUNC). One hundred µl of a luminol (Fluka, Sigma, St. Louis, MO, USA) solution 2 mM in borate buffer (0.2 M pH 9.2) with 2 µg/ml phorbol myristate acetate (PMA, Sigma, St. Louis, MO, USA) were added to each well and the luminescence was measured immediately every 3 min for 1 h at 25 °C in a microplate luminescence reader (Ultra Evolution; Tecan, Männedorf, Zürich, Switzerland). The integral luminescence in relative light units (RLU) was calculated using HBSS++ as a blank.

Plasma lysozyme was measured by a turbidimetric assay as previously described (Sitjà-Bobadilla et al., 2005). Briefly, 5 µl of serum were diluted with 5 µl of 50 mM sodium phosphate buffer pH 6.2 (PB) and incubated with 200 µl of a 0.3 mg/ml *Micrococcus lysodeikticus* (Sigma, St. Louis, MO, USA) suspension in PB. The reduction of the absorbance at 450 nm was determined in a microplate reader (Ultra Evolution; Tecan, Männedorf, Zürich, Switzerland) after 0.5 and 4.5 min and a unit of lysozyme activity was calculated as the amount of enzyme that caused a decrease in absorbance of 0.001 per min.

The alternative complement pathway (ACP) activity was determined using a modification of the method described in Sitjà-Bobadilla et al., (2005) using sheep red blood cells (SRBC; Durviz, Valencia, Spain) as a target. Briefly, 25 µl of a suspension of 2.85 ×10^8^ SRBC/ml in 10 mM EGTA, 10 mM Mg^2+^ HBSS (without calcium and magnesium; Gibco, ThermoFisher Scientific, Waltham, MA, USA) were incubated with 100 µl of different dilutions of serum (1:5, 1:10, 1:20, 1:40, 1:80 and 1:100) in duplicates for 1 h at 20 °C with constant shaking. After spinning down the remaining SRBC, 75 µl of supernatants were transferred to a new 96 well plate and the absorbance at 415 nm was measured. Finally, the dilution of serum that caused 50% hemolysis (CH50) was calculated.

Serum immunoglobulin M (IgM) was analyzed using an ELISA assay. ELISA plates were coated with 50 µl of a 1:6,000 dilution of serum in carbonate/bicarbonate buffer 0.1 M pH 9.6, incubated overnight at 4 °C and, after washing, blocked with 200 µl Tris 20 mM 0.5 M NaCl pH 7.4 (TBS) 5% non-fat dry milk (BioRad, Hercules, CA, USA) for 1 h at 37 °C. Then, plates were washed, incubated with 50 µl of rabbit polyclonal anti-GSB IgM (Palenzuela, Sitjà-Bobadilla & Álvarez Pellitero, 1996) diluted 1:20,000 in TBS, 0.05% Tween 20 and 3% non-fat dry milk (3% TTBS) for 1 h at 37 °C, washed again and incubated for another hour with 50 µl of a 1:1,000 dilution of goat anti-rabbit-horseradish peroxidase (HRP) (Sigma, St. Louis, MO, USA) in 3% TTBS. After careful washing, the reaction was developed using the TMB substrate kit (BioRad, Hercules, CA, USA) following the manufacturer’s instructions, the reaction was stopped after 20 min with 1 N H_2_SO_4_ and the absorbance was measured at 450 nm. Each serum was tested in triplicates and a blank with no serum was included as a background control.

Plasma growth hormone (GH) levels were determined by a homologous GSB radioimmunoassay (RIA) (Martínez-Barberá et al., 1995). The sensitivity and midrange (ED50) of the assay were 0.15 and 2.5–3 ng/ml, respectively. Plasma insulin-like growth factors (IGFs) were extracted by acid-ethanol cryoprecipitation and the concentration of IGF-I was measured using a generic fish IGF-I RIA validated for Mediterranean perciform fish (Vega-Rubín de Celis et al., 2004). The sensitivity and midrange of the assay were 0.05 and 0.7–0.8 ng/ml respectively. Cortisol levels were analyzed using an EIA kit (kit RE52061, IBL International GmbH, Hamburg, Germany) following the manufacturer’s instructions. The limit of detection was 50 pg/ml with an assay midrange of 700 pg/ml.

### Histological analyses

For histological examination, pieces of liver, anterior (immediately after the pyloric caeca) and posterior (immediately before the rectum) intestinal segments were fixed in 10% buffered formalin, embedded in paraffin, 4 µm-sectioned and stained with hematoxylin-eosin (H&E), Giemsa and periodic acid-Schiff (PAS) following standard procedures. For each dietary group, a total of 9 fish were examined (three fish per tank replicate).

### Immunohistochemical analyses

In order to further characterize the observations performed in the histological analyses, 4 µm thick paraffin sections of anterior and posterior intestine samples were collected on Super-Frost-plus microscope slides (Menzel-Gläser, Braunschweig, Germany) and dried overnight. To stain histamine positive cells we used the protocol described in Estensoro et al. (2014) with some modifications. Briefly, samples were deparaffinized and hydrated and the endogenous peroxidase activity was blocked by incubation in methanol:hydrogen peroxide 0.3% v/v in a 9:1 proportion for 40 min. All incubations were performed in a humid chamber, at room temperature and the washing steps consisted of 5 min immersion in TTBS (20 mM Tris–HCl, 0.5 M NaCl, 0.05% Tween 20, pH 7.4) and 5 min immersion in TBS (without Tween 20) unless stated otherwise. Slides were washed and blocked twice for 30 min, first with TBS 5% bovine serum albumin (BSA) and secondly with 1.5% normal goat serum (Vector Laboratories, Burlingame, CA, USA). After washing, they were incubated with a rabbit anti-histamine antibody (Sigma, St. Louis, MO, USA) 1:50 in TBS overnight at 4 °C, washed again and incubated with a biotinylated goat anti-rabbit antibody 1:200 in TBS (Vector Laboratories, Burlingame, CA, USA) for 1 h. The slides were subsequently washed, incubated for 1 h with the avidin-biotin-peroxidase complex (ABC, Vector Laboratories, Burlingame, CA, USA.), washed and developed by incubating with 3,3′-diaminobenzidine tetrahydrochloride chromogen (DAB; Sigma, St. Louis, MO, USA) for 2 min. The reaction was stopped with deionized water and the slides were counterstained for 5 min with Gill’s hematoxylin before being dehydrated and mounted for light microscopy examination.

**Table 2:** Genes included in the PCR-array.

Category	Gene name	Symbol	Accession No.
Energy sensing	*Sirtuin 1*	*sirt1*	KF018666
	*Sirtuin 2*	*sirt2*	KF018667
	*Sirtuin 3*	*sirt3*	KF018668
	*Sirtuin 4*	*sirt4*	KF018669
	*Sirtuin 5*	*sirt5*	KF018670
	*Sirtuin 6*	*sirt6*	KF018671
	*Sirtuin 7*	*sirt7*	KF018672
**Intestinal epithelial barrier**	*Occludin*	*ocln*	KF861990
	*Claudin-12*	*cldn12*	KF861992
	*Claudin-15*	*cldn15*	KF861993
	*Cadherin-1*	*cdh1*	KF861995
	*Cadherin-17*	*cdh17*	KF861996
Enterocyte mass and nutrient absorption	*Intestinal-type alkaline phosphatase*	*alpi*	KF857309
	*Liver-type fatty acid-binding protein*	*fabp1*	KF857311
	*Intestinal fatty acid-binding protein*	*fabp2*	KF857310
	*Ileal fatty acid-binding protein*	*fabp6*	KF857312
Mucus production and goblet cell differentiation	*Mucin 2*	*muc2*	JQ277710
	*Mucin 13*	*muc13*	JQ277713
	*Intestinal mucin*	*i-muc*	JQ277712
	*Transcription factor hes-1-b*	*hes1-b*	KF857344
	*Krueppel-like factor 4*	*klf4*	KF857346
Immunological/inflammatory status	*Tumor necrosis factor-alpha*	*tnfα*	AJ413189
	*Interleukin-1 beta*	*il1β*	AJ419178
	*Interleukin-6*	*il6*	EU244588
	*Interleukin-8*	*il8*	JX976619
	*Interleukin-10*	*il10*	JX976621
	*Cd4*	*cd4*	AM489485
	*Cd8 alpha*	*cd8α*	EU921630
	*Cd8 beta*	*cd8β*	KX231275
	*Galectin-1*	*lgals1*	KF862003
	*Galectin-8*	*lgals8*	KF862004
	*Secreted Immunoglobulin M*	*sIgM*	JQ811851
	*Secreted Immunoglobulin T*	*sIgT*	KX599200
	*Membrane Immunoglobulin M*	*mIgM*	KX599199
	*Membrane Immunoglobulin T*	*mIgT*	KX599201

### RNA extraction, reverse transcription and gene expression analyses

RNA from anterior and posterior intestine samples was extracted using a MagMAX-96 total RNA isolation kit (Life Technologies, Madrid, Spain) as described before (Bermejo-Nogales, Calduch-Giner & Pérez-Sánchez, 2015). The RNA yield was 50–100 µg and RIN (RNA integrity number) values were 8–10 with the Agilent 2100 Bioanalyzer. Reverse transcription (RT) of 500 ng total RNA was performed with random decamers, using the High-Capacity cDNA Archive kit (Applied Biosystems, Foster City, CA, USA) following manufacturer’s instructions. Negative control reactions were performed excluding the reverse transcriptase.

A 96-well PCR array layout was used for the simultaneous profiling under uniform cycling conditions of 35 selected markers of intestinal epithelial barrier, enterocyte mass and nutrient absorption, mucin production and goblet cell differentiation and immunological status (Table 2). The primers were designed to obtain amplicons of 50–150 bp in length (Table S1). Each PCR reaction of 25 µl contained the equivalent of 660 pg of total input RNA, 12.5 µl of 2 × SYBR Green Master Mix (BioRad, Hercules, CA, USA) and 0.9 µM of specific primers. The pipetting and liquid manipulations required to construct the arrays were performed by use of an EpMotion 5070 Liquid Handling Robot (Eppendorf, Hamburg, Germany), and the real-time quantitative PCR was carried out on an Eppendorf Mastercycler Ep Realplex Real-Time PCR Detection System (Eppendorf, Hamburg, Germany). The PCR amplification program consisted of an initial denaturation step at 95 °C for 3 min, followed by 40 cycles of denaturation for 15 s at 95 °C and annealing/extension for 60 s at 60 °C. The efficiency of PCRs (>90%) was checked, and the specificity of reactions was verified by analysis of melting curves (ramping rates of 0.5 °C/10 s over a temperature range of 55–95 °C) and linearity of serial dilutions of RT reactions. Fluorescence data acquired were analyzed using the delta-delta Ct method (Livak & Schmittgen, 2001). Ct values of *β actin* were consistent among groups (with a minimum of 21.88 ± 0.14 and a maximum of 22.21 ± 0.19) in spite of the dietary treatment or the intestine section, and it was used as housekeeping gene. To compare mRNA expression levels, all values were referenced to the expression level of *sirt1* (with a Ct value corresponding to the median of Ct values from all genes analyzed) in the anterior intestine of D1 fish, for which a value 1 was arbitrarily assigned.

### Statistical analyses

Data on growth performance and blood biochemistry of the different experimental diets were analyzed by one-way analysis of variance followed by Holm-Sidak test. Two-way analysis of variance, followed by the Student-Newman-Keuls (SNK) test, was carried out to analyze the effect of dietary treatment on the intestine gene expression (with intestine segment and diet as variable factors). The significance level was set at *P* < 0.05. All analyses were made using the SigmaPlot version 13.0 (Systat Software, San Jose, CA, USA). Hierarchical Clustering to assess the gene expression pattern across intestine between experimental diets was carried out by means of Genesis software (Sturn, Quackenbush & Trajanoski, 2002).

## Results

### Growth performance

To assess the effects of experimental diets on growth performance, data on feed intake, body weight and organo-somatic indexes were calculated (Table 3). Fish fed D1 and D3 diets showed equally good growth performance across the feeding trial, growing from an initial body weight of 29.66 ± 0.49 g to 146.56 ± 1.11 g with overall feed efficiencies of 0.82–0.84 that were not significantly altered by dietary treatment. Conversely, fish fed D2 diet showed increased feed intake and grew significantly faster when compared to the fish fed D1 with a final body weight (167.7 ± 2.26 g) and weight gain (468 ± 6.10%) that were significantly improved by a 12% and 15%, respectively. This yielded specific growth rates that increased significantly from 1.61 ± 0.01 (D1) and 1.58 ± 0.04 (D3) to 1.74 ± 0.01 in fish fed D2 diet.

Organo-somatic indexes calculated as the ratio of tissue to body weight were determined for liver, viscera, mesenteric fat and intestine (Table 3). The resulting viscerosomatic (VSI) and mesenteric (MSI) indexes were not altered by dietary treatment, whereas hepatosomatic index (HSI) of fish fed D2 (2.03 ± 0.06) was significantly higher than those of D1 (1.70 ± 0.06) and D3 (1.77 ± 0.07) fed fish. Dietary intervention did not alter the intestine weight index calculated as the ratio of organ weight to fish weight (IWI). However, when it was determined as the quotient of fish weight to intestine length (ILI), this relative intestine length index was significantly increased from 10.9 ± 1.78 in D1 fish to 19.9 ± 2.12 in fish fed D2. Intermediate ILI values (12.3 ± 2.78), that did not differ significantly from D1 values, were achieved in fish fed D3 diet.

**Table 3:** Effects of experimental diets on growth performance and organo-somatic indexes. Effects of dietary treatment on growth performance of gilthead sea bream juveniles fed to visual satiety with different experimental diets for 14 weeks. Data on body weight, feed intake and growth indices are the mean ±  SEM of triplicate tanks. Data on liver and viscera weight are the mean ± SEM of 18 fish. Data on intestine indexes are the mean ± SEM of nine fish. Different superscript letters in each row indicate significant differences among dietary treatments (Holm-Sidak test *P* < 0.05).

	D1-CTRL	D2-DICOSAN	D3-Probiotic	*P*^1^
Initial body weight (g)	29.2 ± 0.41	29.5 ± 0.14	30.1 ± 0.88	0.149
Final body weight (g)	147.9 ± 1.95^a^	167.7 ± 2.26^b^	145.8 ± 1.82^a^	**0.003**
Final fork length (cm)	17.7 ± 0.17	17.9 ± 0.22	17.4 ± 0.22	0.148
Feed intake (g DM/fish)	148.7 ± 3.48^a^	163.3 ± 3.29^b^	139.9 ± 3.26^a^	**0.003**
CF^2^	2.84 ± 0.07	2.99 ± 0.01	2.87 ± 0.07	0.326
Weight gain (%)^3^	400.0 ± 3.24^a^	468.8 ± 6.10^b^	384.6 ± 18.2^a^	**0.01**
SGR (%)^4^	1.61 ± 0.01^a^	1.74 ± 0.01^b^	1.58 ± 0.04^a^	**0.014**
FE (%)^5^	0.82 ± 0.01	0.85 ± 0.01	0.84 ± 0.01	0.207
Liver weight (g)	2.74 ± 0.11^a^	3.51 ± 0.13^b^	2.69 ± 0.15^a^	**<0.001**
Viscera weight (g)	10.5 ± 0.38^a^	11.9 ± 0.47^b^	10.1 ± 0.35^a^	**0.008**
Mesenteric fat (g)	1.85 ± 0.21	2.30 ± 0.25	2.21 ± 0.32	0.438
Intestine weight (g)	2.65 ± 0.34	2.60 ± 0.19	2.32 ± 0.23	0.631
Intestine length (cm)	11.0 ± 0.81	10.0 ± 0.46	11.6 ± 0.86	0.264
HSI (%)^6^	1.70 ± 0.06^a^	2.03 ± 0.06^b^	1.77 ± 0.07^a^	**0.004**
VSI (%)^7^	6.62 ± 0.15	6.92 ± 0.25	6.78 ± 0.25	0.648
MSI (%)^8^	1.18 ± 0.14	1.36 ± 0.15	1.22 ± 0.12	0.652
IWI (%)^9^	1.59 ± 0.15	1.57 ± 0.09	1.53 ± 0.15	0.951
ILI^10^	10.9 ± 1.78^a^	19.9 ± 2.12^b^	12.3 ± 2.78^ab^	**0.029**

**Notes.**

1 Values resulting from one-way analysis of variance.

2 Condition factor = (100× body weight)/fork length^3^.

3 Weight gain (%) = (100× body weight increase)/initial body weight.

4 Specific growth rate = 100× (ln final body weight − ln initial body weight)/days.

5 Feed efficiency = wet weight gain/dry feed intake.

6 Hepatosomatic index = (100× liver weight)/fish weight.

7 Viscerosomatix index = (100× viscera weight)/fish weight.

8 Mesenteric fat index = (100× mesenteric fat weight)/fish weight.

9 Intestine weight index = (100× intestine weight)/fish weight.

10 Intestine length index = (100× fish weight)/intestine length^3^.

**Table 4:** Effects of experimental diets on blood biochemistry and immunological parameters. Gilthead sea bream juveniles were fed to visual satiety with different experimental diets for 14 weeks. Data are the mean ± SEM of nine fish. Different superscript letters in each row indicate significant differences among dietary treatments (Holm-Sidak test *P* < 0.05).

	D1-CTRL	D2-DICOSAN	D3-Probiotic	*P*^1^
Haemoglobin (g/dl)	10.3 ± 0.24	10.3 ± 0.35	9.6 ± 0.26	0.198
Haematocrit (%)	43.5 ± 1.35	39.6 ± 1.47	38.5 ± 1.82	0.072
Glucose (mg/dl)	36.9 ± 1.38^a^	43.7 ± 2.21^b^	42.5 ± 1.94^ab^	**0.049**
Triglycerides (mM)	6.95 ± 1.30	4.73 ± 1.00	3.48 ± 0.70	0.084
Total cholesterol (mg/dl)	188.5 ± 10.2	191.6 ± 6.48	169.9 ± 6.22	0.130
Total proteins (g/l)	40.5 ± 1.12	40.3 ± 1.22	38.9 ± 1.39	0.629
Antioxidant capacity (Trolox mM)	0.90 ± 0.02	0.88 ± 0.03	0.86 ± 0.02	0.446
GH (ng/ml)	11.4 ± 1.30	10.9 ± 0.99	13.8 ± 1.36	0.157
IGF-I (ng/ml)	83.7 ± 3.4	94.4 ± 6.9	82.7 ± 7.8	0.101
IGF-I/GH	7.73 ± 0.62	8.97 ± 0.56	6.73 ± 0.89	0.250
Respiratory burst (IRLU)	1462.2 ± 239.8^a^	1032.5 ± 197.5^ab^	686.6 ± 182.2^b^	**0.044**
Lysozyme (Units/ml)	93.7 ± 49.7	29.2 ± 11.1	13.4 ± 9.5	0.153
Alternative complement pathway (ACH50)	25.8 ± 5.37	24.5 ± 8.84	29.1 ± 5.18	0.886
IgM (OD 450 nm)	1.27 ± 0.04^a^	1.17 ± 0.06^ab^	1.04 ± 0.09^b^	**0.024**
Cortisol (ng/ml)	8.27 ± 0.96^a^	6.92 ± 0.67^ab^	4.74 ± 1.03^b^	**0.038**

**Notes.**

1 Values resulting from one-way analysis of variance.

### Blood biochemistry and immunological parameters

Different biochemical and immunological analyses were performed on fish blood to assess the effects of experimental diets (Table 4). Dietary intervention did not alter the measured hematological parameters yielding values in the range of 39–43% for Hc and 9.6–10.3 g/dl for Hb concentration. Likewise, plasma levels of triglycerides, cholesterol and proteins were not altered by diet, whereas plasma glucose concentration was significantly increased in fish fed D2 in comparison to D1. Plasma total antioxidant capacity remained unaltered by dietary treatment. A significant diet effect was not found on circulating levels of growth factors (GH, IGF-I), although the highest IGF-I/GH ratio was coincident with the enhanced feed intake and growth found in fish fed D2.

Regarding blood immunological parameters, D3 fed fish showed an anti-inflammatory profile characterized by significantly decreased respiratory burst on PMA stimulated leukocytes and reduced levels of circulating IgM and cortisol when compared to D1 diet. D2 diet yielded intermediate values in all cases. Serum lysozyme values were not significantly different among groups. The alternative complement pathway (ACH50) was completely unaffected by the different diets.

### Histological observations

**Figure 1:**
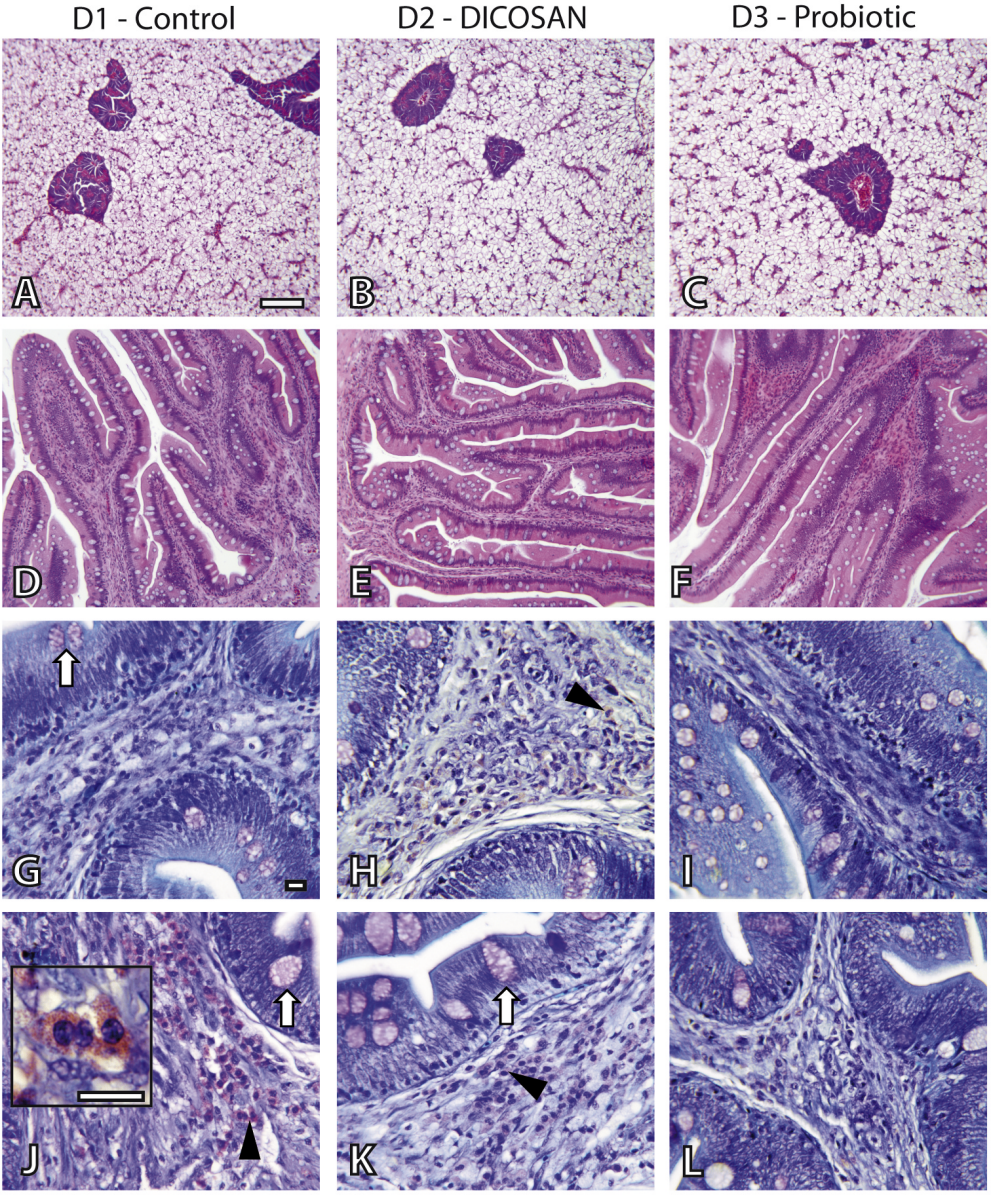
Histological effects of experimental diets. Photomicrographs of liver (A–C), anterior (D–I) and posterior (J–L) intestinal segments of gilthead sea bream fed with D1 (control) (A, D, G, J), D2 (supplemented with DICOSAN) (B, E, H, K) or D3 (supplemented with the probiotic *Bacillus amyloliquefaciens*) (C, F, I, L). Staining, scale bars: A–F = H&E, 100 µm; G–L = Giemsa, 10 µm. White arrows point to goblet cells. Black arrowheads point to submucosa with abundant eosinophilic granulocytes (pink cells). Inset in J (scale bar = 10 µm) shows the characteristic irregular and granular shaped eosinophilic granulocytes.

The effect of the different diets was evaluated by histology. Liver and intestinal samples of all groups were observed to determine differences in general appearance, tissue integrity and cell types present. The liver did not show any pathological effect or significant difference among groups (Figs. 1A–1C). Fat accumulation was similar in the three groups and no steatosis was observed in hepatocytes. Glycogen deposition was similar and moderate in all groups.

In the intestine, goblet cells showed similar histochemical characteristics in all groups and their abundance was slightly higher in the anterior intestine of D3 fed fish (Figs. 1G–1L). Interesting differences were observed in the intestinal architecture of the different dietary groups (Figs. 1D–1F). Anterior intestines of D3 fed fish showed clearly higher intestinal folds or villi, whereas D2 diets induced denser and more complex folds. Another remarkable difference was the presence of eosinophilic granulocytes in the submucosa of the different groups. The irregular shape and size of these cells make exact quantification difficult, thus we only refer to abundance based on overall observations. Eosinophilic granulocytes appeared in almost double amounts in the submucosa of the anterior intestine of fish fed with D2 and were clearly less abundant in the posterior intestine of the D3 group, when compared to the other two groups (Figs. 1G–1L).

### Immunohistochemistry

**Figure 2:**
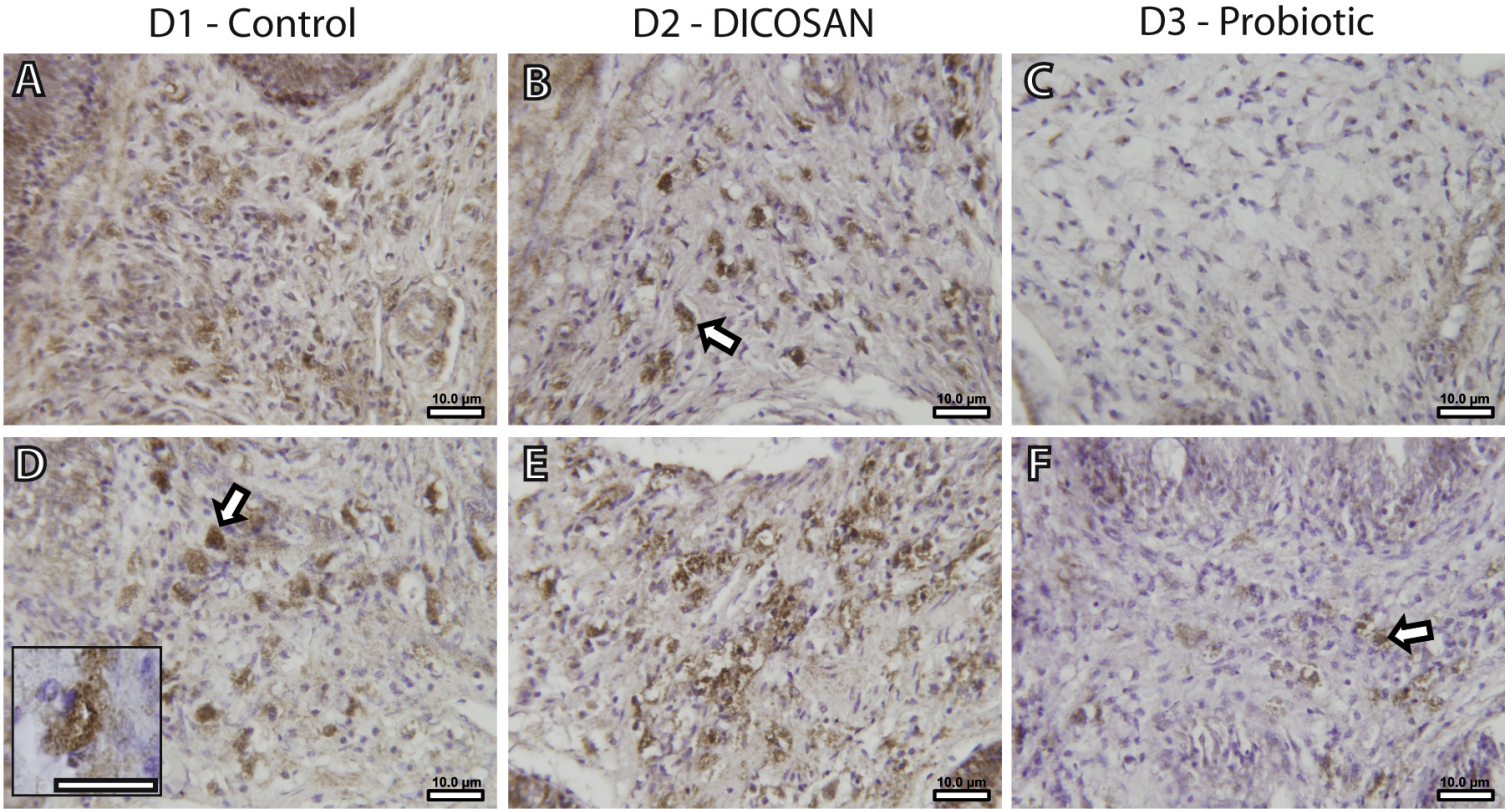
Effects of experimental diets on mast cell abundance in the intestinal submucosa. Representative photomicrographs of anterior (A–C) and posterior (D–F) intestinal segments of gilthead sea bream fed with D1 (control) (A, D), D2 (supplemented with DICOSAN) (B, E) or D3 (supplemented with the probiotic *Bacillus amyloliquefaciens*) (C, F) and stained with an anti-histamine antibody. Histamine positive cells are located in the submucosa and appear brown. All pictures were taken using the same magnification (40×) and the scale bars are 10 µm. White arrows point to some representative mast cells in the submucosa. Inset in D (scale bar = 10 µm) shows a typical histamine positive cell with irregular shape and cytoplasmic granules.

The presence of a differential number of eosinophilic granulocytes in the intestinal submucosa of fish from the different dietary challenges required a further characterization of the cell types. Fish granulocytes differ from those of mammals in their staining properties, which also vary among fish species (Reite & Evensen, 2006). GSB eosinophilic granulocytes can be either acidophils or mast cells, and from these two, only mast cells contain histamine (Mulero et al., 2007). Thus, an anti-histamine antibody was used to further characterize these cells. This allowed detecting histamine-positive cells with similar shape, size and location to those of the observed eosinophilic granulocytes in Figs. 1G–1L (Fig. 2), and therefore we conclude that at least part of the observed eosinophilic granulocytes were histamine-positive mast cells. As stated before, reliable quantification of these cells could not be performed, thus we only refer to significant changes that can be observed by microscopic observation. The anterior intestine of D1 and D2 fed fish had large numbers of mast cells, whereas at least half the amount was found in D3 fed fish (Figs. 2A–2C). In the posterior intestine, mast cells were more abundant in D2 fed fish and clearly lower in D3 fed fish when compared to the control animals (Figs. 2D–2F). Although the presence of another type of eosinophilic granulocytes (acidophils) cannot be discarded, it is clear that, at least, the mast cell population in the intestinal submucosa is affected by the different dietary treatments.

**Table 5:** Relative gene expression of anterior and posterior intestinal sections of gilthead sea bream fed experimental diets. Data are the mean ± SEM of 9 fish. All values are referred to *sirt1* with an arbitrary value of 1 in the anterior intestine of fish fed D1. *P*-values are the result of two-way analysis of variance followed by SNK test.

	Anterior intestine			Posterior intestine			ANOVA II, *P*-value		
	D1-CTRL	D2-DICOSAN	D3-Probiotic	D1-CTRL	D2-DICOSAN	D3-Probiotic	Int. Segment	Diet	Interaction
*sirt1*	1.03 ± 0.09	0.84 ± 0.04^∗^	0.77 ± 0.04^∗^	0.85 ± 0.09	0.84 ± 0.09	0.89 ± 0.15	0.847	0.441	0.283
*sirt2*	1.22 ± 0.11	1.05 ± 0.03^∗^	0.93 ± 0.06^∗^	1.09 ± 0.10	1.06 ± 0.11	1.12 ± 0.13	0.741	0.378	0.253
*sirt3*	0.52 ± 0.07	0.35 ± 0.02^∗^	0.34 ± 0.03^∗^	0.46 ± 0.08	0.42 ± 0.04	0.52 ± 0.08	0.187	0.203	0.129
*sirt4*	0.15 ± 0.02	0.13 ± 0.01	0.12 ± 0.01	0.13 ± 0.02	0.12 ± 0.02	0.13 ± 0.02	0.549	0.642	0.569
*sirt5*	1.08 ± 0.10	1.04 ± 0.05	0.96 ± 0.10	1.11 ± 0.12	1.04 ± 0.058	1.24 ± 0.09	0.171	0.768	0.264
*sirt6*	0.24 ± 0.03	0.21 ± 0.01	0.20 ± 0.01	0.21 ± 0.03	0.19 ± 0.02	0.25 ± 0.03	0.939	0.465	0.162
*sirt7*	0.26 ± 0.02	0.24 ± 0.01†	0.18 ± 0.01^∗∗^	0.25 ± 0.02	0.23 ± 0.02	0.26 ± 0.05	0.360	0.453	0.219
***ocln***	6.36 ± 0.66	6.02 ± 0.23	6.73 ± 0.61	8.52 ± 0.54	10.2 ± 0.83	9.71 ± 1.35	<**0.001**	0.583	0.431
***cldn12***	0.94 ± 0.10	1.05 ± 0.05	0.95 ± 0.04	1.02 ± 0.12	1.29 ± 0.13	1.16 ± 0.13	**0.035**	0.154	0.716
***cldn15***	27.3 ± 3.69	23.5 ± 0.76	26.5 ± 1.95	40.1 ± 3.73	48.7 ± 6.90	48.7 ± 9.13	<**0.001**	0.772	0.493
***cdh1***	22.8 ± 2.76	14.4 ± 0.68^∗∗^	14.8 ± 0.77^∗∗^	12.22 ± 1.17	15.4 ± 1.63	12.5 ± 0.92	**0.001**	**0.032**	**0.001**
***cdh17***	72.0 ± 7.72	62.7 ± 1.77	71.9 ± 4.14	45.9 ± 2.07	53.03 ± 4.42	49.9 ± 4.06	<**0.001**	0.759	0.150
*alpi*	112.7 ± 9.78	124.4 ±7.99†	97.7 ± 8.36	28.8 ± 3.79	40.8 ± 6.60	35.5 ± 3.35	<**0.001**	**0.041**	0.313
*fabp1*	159.5 ± 16.5	155.4 ± 8.64	147.8 ± 6.87	119.8 ± 18.3	136.6 ± 30.2	142.4 ± 23.8	0.113	0.882	0.872
*fabp2*	364.0 ± 94.9	734.4 ± 104.9^∗^	556.2 ± 73.8	606.9 ± 83.5	1208.5 ± 210.6^∗^	799.2 ± 189.8	**0.006**	**0.003**	0.599
*fabp6*	<0.05	<0.05	<0.05	3423.5 ± 507.1	2563.6 ± 440.6	2874.4 ± 435.9	<**0.001**	0.424	0.424
*muc2*	41.5 ± 3.3	44.4 ± 3.03	39.06 ± 2.60	37.9 ± 3.24	32.6 ± 2.84	39.5 ± 4.02	0.063	0.923	0.142
*muc13*	149.2 ± 17.1	118.7 ± 5.49	107.9 ± 6.22^∗^	101.6 ± 9.47	121.7 ± 10.2	98.7 ± 7.93	**0.027**	0.067	**0.037**
*i-muc*	0.14 ± 0.02	0.15 ± 0.04	0.13 ± 0.02	7.29 ± 3.10	26.6 ± 16.9	29.1 ± 18.2	**0.015**	0.513	0.513
*hes1-b*	3.82 ± 0.67	3.3 ± 0.36	3.59 ± 0.39	4.04 ± 0.45	4.61 ± 0.70	5.05 ± 1.17	0.078	0.808	0.636
*klf4*	2.88 ± 0.25	2.68 ± 0.17	3.15 ± 0.28	2.21 ± 0.18	2.90 ± 0.19^∗^	2.87 ± 0.22^∗^	0.186	0.136	0.143
*tnfα*	0.13 ± 0.02	0.11 ± 0.01	0.12 ± 0.01	0.12 ± 0.01	0.14 ± 0.01	0.16 ± 0.02	0.094	0.356	0.108
*il1β*	0.08 ± 0.01	0.08 ± 0.01	0.09 ± 0.01	0.11 ± 0.01	0.12 ± 0.01	0.12 ± 0.02	**0.002**	0.849	0.964
*il6*	0.03 ± 0.01	0.02 ±0.00†	0.04 ± 0.00	0.02 ± 0.00	0.02 ± 0.00	0.04 ± 0.01	0.615	**0.024**	0.596
*il8*	0.25 ± 0.04	0.13 ± 0.01^∗∗^	0.17 ± 0.03	0.32 ± 0.09	0.21 ± 0.03	0.22 ± 0.04	**0.050**	**0.038**	0.939
*il10*	0.11 ± 0.02	0.12 ± 0.01	0.11 ± 0.01	0.16 ± 0.01	0.16 ± 0.02	0.17 ± 0.03	**0.001**	0.916	0.817
*cd4*	0.53 ± 0.12	0.35 ± 0.02^∗^	0.32 ± 0.02^∗^	0.55 ± 0.04	0.52 ± 0.06	0.45 ± 0.05	**0.032**	**0.038**	0.446
*cd8α*	0.80 ± 0.09	0.86 ± 0.09	0.71 ± 0.05	0.80 ± 0.13	1.10 ± 0.15	1.15 ± 0.19	**0.036**	0.382	0.259
*cd8β*	0.12 ± 0.02	0.13 ± 0.02	0.10 ± 0.01	0.14 ± 0.02	0.18 ± 0.03	0.18 ± 0.04	**0.008**	0.522	0.458
*lgals1*	5.70 ± 0.70	5.92 ± 0.37	5.31 ± 0.50	10.2 ± 0.97	10.4 ± 0.83	9.86 ± 1.23	<**0.001**	0.741	1.000
*lgals8*	3.77 ± 0.40	2.76 ± 0.15^∗^	2.72 ± 0.24^∗^	3.00 ± 0.33	4.23 ± 0.70	3.92 ± 0.78	0.131	0.935	0.074
*sIgM*	10.1 ± 3.59	4.14 ± 0.86	5.77 ± 0.66	11.9 ± 3.87	8.09 ± 2.90	8.36 ± 1.67	0.180	0.134	0.906
*sIgT*	0.10 ± 0.03	0.08 ± 0.01†	0.12 ± 0.02	0.05 ± 0.02	0.08 ± 0.02	0.12 ± 0.04	0.403	0.146	0.518
*mIgM*	0.20 ± 0.07	0.15 ± 0.02	0.13 ± 0.01	0.40 ± 0.09	0.23 ± 0.04	0.38 ± 0.10	**0.002**	0.231	0.41
*mIgT*	0.45 ± 0.03	0.44 ± 0.05	0.58 ± 0.11	0.59 ± 0.06	0.83 ± 0.09	1.56 ± 0.46^∗^	<**0.001**	**0.004**	**0.038**

**Notes.**

Asterisks (*), indicate significant differences (^∗^*P* < 0.05,^∗∗^*P* < 0.01) between control and experimental diets. Crosses (†), indicate significant differences (†*P* < 0.05) between experimental diets.

### Gene expression profiles

The gene expression profile of selected markers was studied in samples of anterior and posterior intestines of fish fed the different experimental diets in order to characterize the general intestinal status of these fish. For most studied genes, a clear expression pattern across intestinal segments was found, regardless of the diet. The structural genes *cdh1, cdh17,* had higher expression in anterior intestine, whereas *cldn15, ocln* and *cldn12*, were mostly expressed in posterior intestine (as indicated by the low *P* values in the intestinal segment column of Table 5). Markers of enterocyte mass and nutrient absorption were also differentially expressed across the intestine, with higher expression of *alpi* in the anterior intestine and *fabp6* in the posterior intestine. Some markers of mucus production (*muc13*, *i-muc*) also showed a pronounced spatial pattern. Markers of immunological status (*il1 β*, *il8*, *il10*, *cd4*, *cd8 α*, *cd8 β*, *lgals1*, *mIgM* and *mIgT*) were significantly higher expressed in posterior intestine.

Regarding the diet effect, fish fed D2 and D3 diets showed a reduced expression of energy sensing molecules (*sirt1*, *sirt2*, *sirt3*), *cdh1*, *cd4* and *lgals8* in anterior intestine, compared to fish fed D1 diet. Fish fed D2 diet showed the highest expression of *alpi*, significantly in the anterior intestine when compared to D3 fed fish. The expression of *fabp2* was higher in D2 than in D1 fish in both anterior and posterior intestinal segments, whereas intermediate values were found in D3 fish. Overall, the expression level of mucin genes was not significantly altered by diet, although the diet × intestine section interaction was statistically significant for *muc13*, and its expression was significantly down-regulated in D3 fish in comparison to D1 fish. Regarding the named *i-muc* gene*,* a diet effect was not statistically significant for this exclusive fish gene that was mostly expressed in the posterior intestine of GSB. D2 induced a significant down-regulation of *il8* in anterior intestine, and both D2 and D3 diets down-regulated the expression of *cd4* and *lgals8* in the anterior intestine segment. D3 diet did not modify significantly the expression of membrane and secreted IgM isoforms, although this diet significantly increased the expression of the membrane *IgT* (*mIgT*) in posterior intestine.

**Figure 3:**
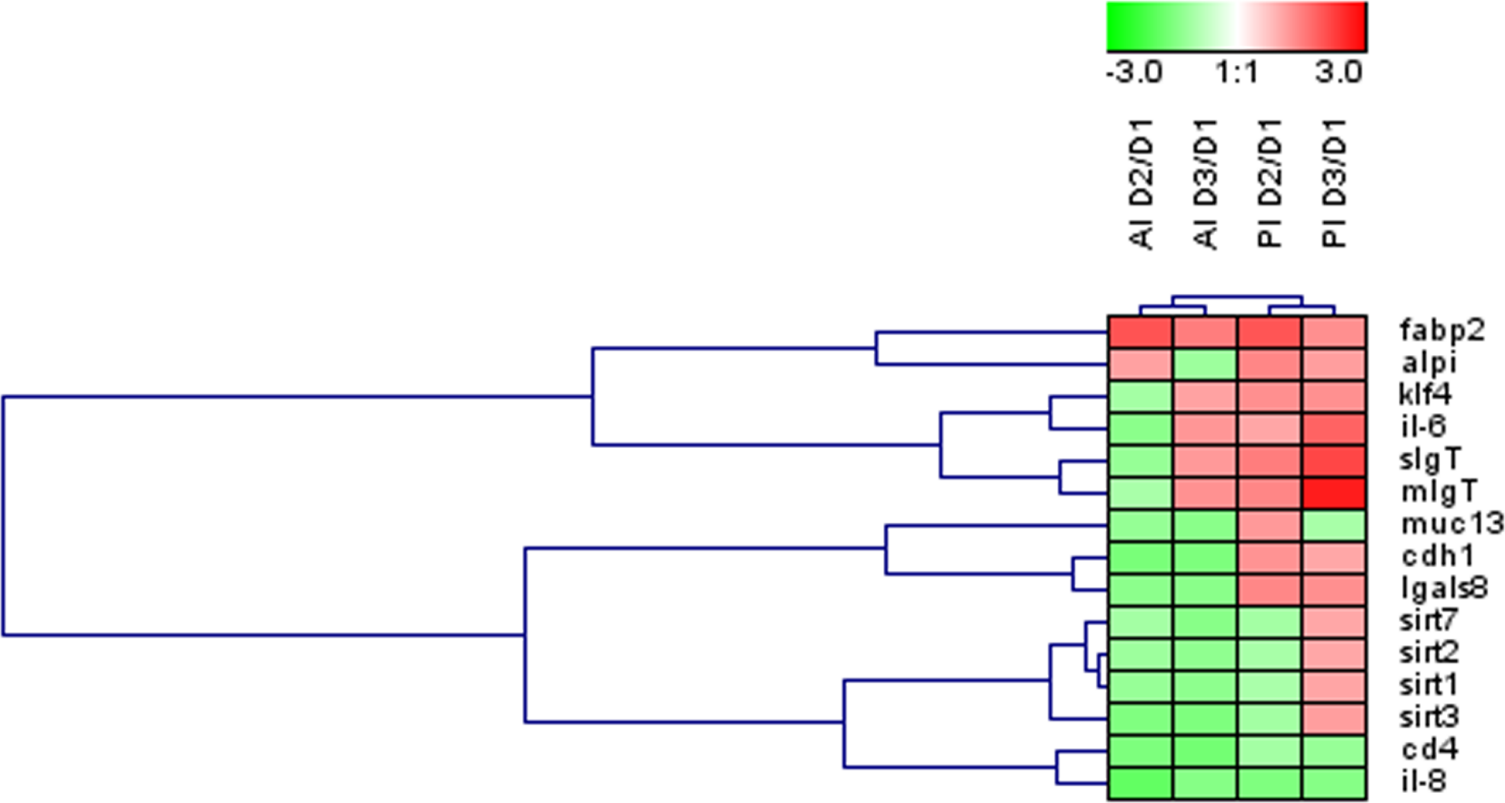
Effects of experimental diets on genes related to intestinal architecture, absorption and immune response. Hierarchical heatmap of fold-changes (experimental group (D2, D3) *vs.* control (D1)) for differentially expressed genes in at least one of the intestinal sections. AI, anterior intestine; PI, posterior intestine.

Globally across the intestine, the dietary-mediated effects affected the expression of 15 out of the 35 genes studied (43%). The heatmap in Fig. 3 was constructed with the expression values of those differentially regulated genes in order to obtain a visual overview of the changes. Fish fed D2 diet revealed an overall down-regulation of genes related to intestinal integrity (*cdh1*) and energy sensing (*sirt1-3*) mainly affecting the anterior intestine. Fish fed D3 diet showed more prominent effects on posterior intestine with up-regulation of genes related to immune response (*IgT, il6*).

## Discussion

Evidence in mammals indicates that the metabolic utilization of ingested nutrients determines satiety and feeding behavior (Scharrer & Langhans, 1986). Since MCFAs can enter directly in the portal vein and their transport across the mitochondrial membrane does not require carnitine palmitoyltransferase-1, their final tissue uptake and oxidation is accelerated, acting as satiety factors. Hence, in comparison to long-chain triglycerides, preloading of oral emulsions of medium-chain triglycerides decreased feed intake in rats (Ooyama et al., 2009). High dietary inclusion levels of medium-chain triglycerides (15–30%) also decreased feed intake in polka-dot grouper (*Cromileptes altivelis*) (Williams et al., 2006), and a strong inverse relationship between dietary medium-chain triglycerides (0.4–15%) and feed intake was found in Atlantic salmon (*Salmo salar*) (Nordrum et al., 2003). By contrast, feed intake was not affected by medium-chain triglyceride supplementation (5–15%) in sunshine bass (female white bass *Morone chrysops* × male striped bass *Morone saxatilis*) (Trushenski, 2009) and rainbow trout (*Oncorhynchus mykiss*) (Figueiredo-Silva et al., 2012), which indicates that other factors, besides metabolic fuel availability, regulate feed intake in fish. Indeed, our results showed that dietary supplementation with MCFAs in the form of a sodium salt of coconut fatty acid distillate (D2 diet) was able to enhance the overall feed intake and growth rates of GSB with no signs of histopathological damage in liver and intestine tissues. This growth-promoting action was supported by histological and gene expression data at the intestine level (see below) and by a slight, although non-significant, increase of the circulating IGF/GH quotient, which can be viewed as an increased liver GH-responsiveness to the anabolic action of GH. This agrees with the observation that dietary medium-chain triglycerides act in young and growing pigs via stimulation of somatotropic endocrine pathways, minimizing weaning-associated disorders such as slow growth and diarrhea (Miller et al., 2016). Other potential benefits could be mediated by changes in the intestinal microbiota (Lai et al., 2014), although the therapeutic potential of MCFAs and their potential benefits on feed intake and key performance indicators seems to be highly dependent on the intake dose and age of piglets (Lai et al., 2014: Zhang et al., 2016). In this regard, it is noteworthy that the gene expression of several sirtuins was down-regulated in the anterior intestine of fish fed D2 or D3 diets. Since sirtuins are NAD^+^-dependent deacetylases that act as energy sensors, it can be argued that both D2 and D3 diets regulate energy metabolism at the intestinal local level by means of epigenetic-related mechanisms. Recently, the tissue-specific regulation of the seven mammalian sirtuin counterparts of GSB has been described (Simó-Mirabet et al., 2017). The present study provides further evidence for a functional regulation of sirtuins at intestinal level in farmed fish, which can be viewed as a consequence of a reduced energy demand in fish with an increased feed intake (D2 diet) or as a part of an adaptive hypo-metabolic conditioning (D3 diet) in fish with a slight reduction of feed intake.

Absorption of lipids occurs mostly in the first segments of fish intestines, where bile salts are secreted to hydrolyze triglycerides to free fatty-acids and glycerol (Sundell & Rønnestad, 2011) resembling mammalian small intestines. In accordance, expression of *fabp6*, involved in reabsorption of bile acids and exclusively expressed in mammalian ileums (Besnard et al., 2002), can only be found in the posterior intestinal segment of GSB and other fish species (Alves-Costa et al., 2008; Pérez-Sánchez et al., 2015; Calduch-Giner, Sitjà-Bobadilla & Pérez-Sánchez 2016). FABP2, involved in regulation of the intracellular concentration of free fatty acids, preferentially binds long-chain fatty acids (LCFAs) (Lowe et al., 1987), but can also bind MCFAs (Huang et al., 2002). Several studies in mammals associated FABP2 expression or genetic variants to insulin resistance and type 2 diabetes in individuals fed different sources of MCFAs or LCFAs (Rubin et al., 2012). Our results show a clear up-regulation of *fabp2* and an increase in plasma glucose levels upon MCFA intake (D2 diet). In agreement with the current results, rainbow trout fed high levels of coconut oil also showed increased plasma glucose levels (Figueiredo-Silva et al., 2012). Thus, there is a clear relationship among lipid source, *fabp2* expression and glucose tolerance both in mammals and teleost fish, and the underlying mechanisms deserve further study.

In our study, additional changes associated to coconut oil supplementation are the increase in intestinal complexity and gene expression of *alpi* in anterior intestine, which are related to intestinal architecture and nutrient absorption. In fish, a decrease in Alpi activity has been linked to malnutrition (Bakke-McKellep et al., 2000; Ducasse-Cabanot et al., 2007). Conversely, unchanged Alpi activity or gene expression levels in intestine were related with good growth performance values (Estensoro et al., 2016). In the current study, MCFAs (D2 diet) increased *alpi* gene expression which, together with the increased complexity of intestinal folds in the main absorptive intestinal segment, can be directly related to the increased intake and growth performance of this dietary group. Moreover, the intestine of D2 fed fish was shorter than that of the other two experimental groups. This trend was also found when comparing carnivorous with omnivores and herbivorous fish, since the total intestinal surface required for the absorption of nutrients is directly linked to digestive efficiency, increasing gut length allometrically with the body length (Karachle & Stergiou, 2010).

A recent study performed in mice also showed that MCFA addition to fish oil formulations is able to induce an anti-inflammatory profile, decreasing the host response to inflammatory challenge (Carlson et al., 2015). In view of these results, MCFA supplementation has been proposed as a candidate to formulate optimized diets to be used as therapeutic intervention for diseases derived from chronic inflammation. Coconut oil as a source of MCFA in GSB (D2 diet) induced no significant changes in serum lysozyme, complement activity, IgM levels or respiratory burst of stimulated blood leukocytes. However, significant down-regulation of *cd4*, *il8* and *lgals8* was observed in the intestine, hinting to some anti-inflammatory effects on GSB intestinal immunity. By contrast, an increase in eosinophilic granulocytes, particularly in mast cells, was observed in these intestines. Nonetheless, the lack of any prototypical pro-inflammatory signal, such as *tnf α* or *il1 β* up-regulation, suggests that these cells have been recruited and reside inactive in the intestinal submucosa as a kind of surveillance machinery ready to be activated in case of threat. Although in the present study no challenge was performed to evaluate the readiness of the immune system of GSB upon pathogen exposure, there is evidence that coconut oil supplementation can increase survival of catfish upon *Aeromonas hydrophila* challenge (Vargas et al., 2013).

Probiotics modulate the gastrointestinal microbial communities having diverse effects, such as inhibition or suppression of pathogen growth, improvement of stress tolerance, stimulation of growth or modulation of the host immune status (Balcázar et al., 2006; Wang, Li & Lin, 2008). Accordingly, oral administration of *B. amyloliquefaciens* in mice showed a protective effect against *Clostridium difficile* and has been proposed to be used in humans (Geeraerts et al., 2015). It also showed alleviating effects on immune stress induced by lipopolysaccharide (LPS) challenge in broilers, by ameliorating the growth performance and inducing an anti-inflammatory shift down-regulating pro-inflammatory gene expression and increasing *IL10* expression (Li et al., 2015). Its use in aquaculture has also been proposed, and *B. amyloliquefaciens* showed potential to control vibriosis in turbot (*Scophthalmus maxima*) (Chen et al., 2016a) and other fish pathogens (Chen et al., 2016b). The present study does not deal with any pathological challenge, but aims to define the effect of this probiotic administration on the basal health of GSB in order to assess their readiness to face a threat. In addition, the *Bacillus*-based probiotic-supplemented diet (D3) did not induce negative effects on the growth performance of GSB. Likewise, beneficial effects upon infection, but no interference with growth, have been documented in similar trials with different *Bacillus* species in Nile tilapia (Aly, Mohamed & John, 2008) and rainbow trout (Raida et al., 2003). Many studies have demonstrated the beneficial effects of *B. amyloliquefaciens* on broiler performance parameters (Sánchez et al., 2006; Mallo et al., 2010), but in some cases, probiotics supplementation might not affect growth parameters, and a protective effect on growth can only be observed upon challenge (Li et al., 2015).

Our results with fast growing juveniles of GSB also showed clear interesting outcomes of the *Bacillus*-based probiotic (D3 diet) on intestinal architecture and mucus production. Anterior intestine showed higher intestinal folds in agreement with the results obtained in Nile tilapia fed the same probiotic bacteria (Silva et al., 2015). In addition, D3 diet correlated with low numbers of goblet cells and down-regulation of the expression of the transmembrane *muc13*, a major enterocyte component of GSB (Pérez-Sánchez et al., 2013). It must be argued that *muc13* does not contribute significantly to the extended glycocalyx of GSB when the mucin secretion from goblet cells remains high. Otherwise, studies on Muc13-/- mice suggest that this mucin has an anti-inflammatory function and anti-apoptotic effects in epithelial cells (Sheng et al., 2011; Sheng et al., 2013). In this regard, in our experimental model, the down-regulation of *muc13* could be viewed as a consequence rather than a cause of an overall anti-inflammatory status, which comprised a decrease in *lgals8* and *cd4* transcripts in anterior intestine, a lower respiratory burst activity of blood leukocytes, lower amounts of circulating IgM and cortisol, and lower numbers of eosinophilic granulocytes, in particular mast cells, in the intestinal submucosa.

The anti-inflammatory effects of *B. amyloliquefaciens* as a probiotic have been already reported in birds and mammals. This probiotic ameliorated the damage caused by inflammation in dextran sulphate sodium (DSS)-induced colitis in mice (Hairul Islam et al., 2011) and LPS-induced stress in broilers (Li et al., 2015) by down-regulating expression of pro-inflammatory cytokines such as *TNF α*, *IL1 β* and *IL2* and up-regulating the anti-inflammatory cytokine *IL10* upon exacerbated inflammation. In addition, a decrease in neutrophil myeloperoxidase and an increase in antioxidant molecules (superoxide dismutase and catalase) was observed (Hairul Islam et al., 2011). In the present study, superoxide dismutase and catalase were not measured, but the observed decrease in oxidative radicals measured with the respiratory burst assay indirectly reflects this anti-oxidant effect. A similar decrease in respiratory burst was observed in European sea bass fed a different probiotic, which also induced an anti-oxidant status (Guardiola et al., 2016). Opposite to the decrease in circulating levels of IgM, a significant up-regulation of *mIgT* expression was observed in posterior intestine. IgT is the key mucosal immunoglobulin in teleosts (Zhang et al., 2010) and the importance of its fine regulation upon infection in GSB intestines has been recently described (Piazzon et al., 2016). The presence of higher number of *mIgT* transcripts can be related to higher numbers of IgT^+^ B cells. The availability of this larger pool of IgT^+^ B cells can be critical upon infection or any other threat, as these cells are already in the local environment ready to be activated and exert their function as effector cells. In fact, when the transcription of *IgT* in GSB intestines is impaired, as it happens when fish are fed diets with high plant ingredient substitution, the disease outcome upon parasitic infection is worse (Piazzon et al., 2016). The results of the current study with *B. amyloliquefaciens* CECT 5940 supplementation as a probiotic (D3 diet) point to a fine-tuning of the mucosal immunity by increasing the number of resident mucosal related IgT^+^ B cells to improve the surveillance mechanisms in the posterior intestine, where intestinal immune responses mainly take place.

## Conclusions

Altogether, we observed differential beneficial effects induced by these dietary supplements that are related to their different nature. MCFAs induced positive effects on GSB growth and intestinal architecture mainly affecting the anterior intestinal segment where absorption mostly takes place. Conversely, *B. amyloliquefaciens* CECT 5940 supplementation had key effects in the regulation of the immune status inducing anti-inflammatory and anti-oxidant effects that can potentially be advantageous upon infection or stressful situations. Separately, the two additives showed interesting effects posing a promising way to improve health and disease resistance in aquaculture.

##  Supplemental Information

10.7717/peerj.4001/supp-1 Table S1 Forward and reverse primers used for real-time qPCR

10.7717/peerj.4001/supp-2 Data S1 Raw data

## Additional Information and Declarations

### Data Availability

The following information was supplied regarding data availability: The raw data has been provided as a Supplemental File.

## References

[ref-1] Abriouel H, Franz CMAP, Omar NB, Galvez A (2011). Diversity and applications of *Bacillus* bacteriocins. FEMS Microbiology Reviews.

[ref-2] Alves-Costa FA, Denovan-Wright EM, Thisse C, Thisse B, Wright JM (2008). Spatio-temporal distribution of fatty acid-binding protein 6 (fabp6) gene transcripts in the developing and adult zebrafish (*Danio rerio*). FEBS Journal.

[ref-3] Aly SM, Mohamed MF, John G (2008). Effect of probiotics on the survival, growth and challenge infection in Tilapia nilotica (*Oreochromis niloticus*). Aquaculture Research.

[ref-4] Bakke-McKellep AM, McL Press C, Baeverfjord G, Krogdahl Å, Landsverk T (2000). Changes in immune and enzyme histochemical phenotypes of cells in the intestinal mucosa of Atlantic salmon, *Salmo salar* L., with soybean meal-induced enteritis. Journal of Fish Diseases.

[ref-5] Balcázar JL, Blas I, Ruiz-Zarzuela I, Cunningham D, Vendrell D, Múzquiz JL (2006). The role of probiotics in aquaculture. Veterinary Microbiology.

[ref-6] Benedito-Palos L, Ballester-Lozano GF, Simó P, Karalazos V, Ortiz Á, Calduch-Giner J, Pérez-Sánchez J (2016). Lasting effects of butyrate and low FM/FO diets on growth performance, blood haematology/biochemistry and molecular growth-related markers in gilthead sea bream (*Sparus aurata*). Aquaculture.

[ref-7] Bergsson G, Arnfinnsson J, Steingrímsson Ó, Thormar H (2001). Killing of Gram-positive cocci by fatty acids and monoglycerides. APMIS.

[ref-8] Bermejo-Nogales A, Calduch-Giner JA, Pérez-Sánchez J (2015). Unraveling the molecular signatures of oxidative phosphorylation to cope with the nutritionally changing metabolic capabilities of liver and muscle tissues in farmed fish. PLOS ONE.

[ref-9] Besnard P, Niot I, Poirier H, Clément L, Bernard A (2002). New insights into the fatty acid-binding protein (FABP) family in the small intestine. Molecular and Cellular Biochemistry.

[ref-10] Calduch-Giner JA, Sitjà-Bobadilla A, Pérez-Sánchez J (2016). Gene expression profiling reveals functional specialization along the intestinal tract of a carnivorous teleostean fish (*Dicentrarchus labrax*). Frontiers in Physiology.

[ref-11] Carlson SJ, Nandivada P, Chang MI, Mitchell PD, O’Loughlin A, Cowan E, Gura KM, Nose V, Bistrian B, Puder M (2015). The addition of medium-chain triglycerides to a purified fish oil-based diet alters inflammatory profiles in mice. Metabolism: Clinical and Experimental.

[ref-12] Cerezuela R, Guardiola FA, Cuesta A, Esteban MA (2016). Enrichment of gilthead seabream (*Sparus aurata* L.) diet with palm fruit extracts and probiotics: effects on skin mucosal immunity. Fish and Shellfish Immunology.

[ref-13] Chen Y, Li J, Xiao P, Li GY, Yue S, Huang J, Zhu WY, Mo ZL (2016a). Isolation and characterization of *Bacillus* spp. M001 for potential application in turbot (*Scophthalmus maximus* L) against *Vibrio anguillarum*. Aquaculture Nutrition.

[ref-14] Chen Y, Li J, Xiao P, Zhu W, Mo ZL (2016b). The ability of marine Bacillus spp. isolated from fish gastrointestinal tract and culture pond sediment to inhibit growth of aquatic pathogenic bacteria. Iranian Journal of Fisheries Sciences.

[ref-15] Das A, Nakhro K, Chowdhury S, Kamilya D (2013). Effects of potential probiotic *Bacillus amyloliquifaciens* FPTB16 on systemic and cutaneous mucosal immune responses and disease resistance of catla (*Catla catla*). Fish and Shellfish Immunology.

[ref-16] Diaz D (2007). Effect of *Bacillus amyloliquefaciens* CECT-5940 spores on broiler performance and digestibility. http://en.engormix.com/MA-poultry-industry/articles/effect-bacillus-amyloliquefaciens-cect5940-t795/p0.htm.

[ref-17] Done HY, Venkatesan AK, Halden RU (2015). Does the recent growth of aquaculture create antibiotic resistance threats different from those associated with land animal production in agriculture?. AAPS Journal.

[ref-18] Ducasse-Cabanot S, Zambonino-Infante J, Richard N, Medale F, Corraze G, Mambrini M, Robin J, Cahu C, Kaushik S, Panserat S (2007). Reduced lipid intake leads to changes in digestive enzymes in the intestine but has minor effect on key enzymes of hepatic intermediary metabolism in rainbow trout (*Oncorhynchus mykiss*). Animal.

[ref-19] Estensoro I, Ballester-Lozano G, Benedito-Palos L, Grammes F, Martos-Sitcha JA, Mydland L-T, Calduch-Giner JA, Fuentes J, Karalazos V, Á Ortiz., Øverland M, Sitjà-Bobadilla A, Pérez-Sánchez J (2016). Dietary butyrate helps to restore the intestinal status of a marine teleost (*Sparus aurata*) fed extreme diets low in fish meal and fish oil. PLOS ONE.

[ref-20] Estensoro I, Mulero I, Redondo MJ, Alvarez-Pellitero P, Mulero V, Sitjà-Bobadilla A (2014). Modulation of leukocytic populations of gilthead sea bream (*Sparus aurata*) by the intestinal parasite *Enteromyxum leei* (Myxozoa: Myxosporea). Parasitology.

[ref-21] FAO (2016). The state of world fisheries and aquaculture. Contributing to food security and nutrition for all. Rome. http://www.fao.org/3/a-i5555e.pdf.

[ref-22] Figueiredo-Silva C, Kaushik S, Terrier F, Schrama JW, Médale F, Geurden I (2012). Link between lipid metabolism and voluntary food intake in rainbow trout fed coconut oil rich in medium-chain TAG. British Journal of Nutrition.

[ref-23] Geeraerts S, Ducatelle R, Haesebrouck F, Van Immerseel F (2015). *Bacillus amyloliquefaciens* as prophylactic treatment for *Clostridium difficile*-associated disease in a mouse model. Journal of Gastroenterology and Hepatology.

[ref-24] Guardiola FA, Porcino C, Cerezuela R, Cuesta A, Faggio C, Esteban MA (2016). Impact of date palm fruits extracts and probiotic enriched diet on antioxidant status, innate immune response and immune-related gene expression of European seabass (*Dicentrarchus labrax*). Fish and Shellfish Immunology.

[ref-25] Hanczakowska E, Świątkiewicz M, Natonek-Wiśniewska M, Okoń K (2016). Medium chain fatty acids (MCFA) and/or probiotic *Enterococcus faecium* as a feed supplement for piglets. Livestock Science.

[ref-26] Hairul Islam VI, Prakash Babu N, Pandikumar P, Ignacimuthu S (2011). Isolation and characterization of putative probiotic bacterial strain, *Bacillus amyloliquefaciens*, from north east Himalayan soil based on *in vitro* and *in vivo* functional properties. Probiotics and Antimicrobial Proteins.

[ref-27] Huang H, Starodub O, McIntosh A, Kier AB, Schroeder F (2002). Liver fatty acid-binding protein targets fatty acids to the nucleus. Real time confocal and multiphoton fluorescence imaging in living cells. The Journal of Biological Chemistry.

[ref-28] Karachle PK, Stergiou KI (2010). Gut length for several marine fish: relationships with body length and trophic implications. Marine Biodiversity Records.

[ref-29] Khan SH, Iqbal J (2016). Recent advances in the role of organic acids in poultry nutrition. Journal of Applied Animal Research.

[ref-30] Lai WK, Yen HC, Lin CS, Chiang SH (2014). The effects of dietary medium-chain triacylglycerols on growth performance and intestinal microflora in young pigs. Journal of Animal and Feed Sciences.

[ref-31] Li Y, Zhang H, Chen YP, Yang MX, Zhang LL, Lu ZX, Zhou YM, Wang T (2015). *Bacillus amyloliquefaciens* supplementation alleviates immunological stress and intestinal damage in lipopolysaccharide-challenged broilers. Animal Feed Science and Technology.

[ref-32] Livak KJ, Schmittgen TD (2001). Analysis of relative gene expression data using real-time quantitative PCR and the 2(-Delta Delta C(T)) Method. Methods.

[ref-33] Lowe JB, Sacchettini JC, Laposata M, McQuillan JJ, Gordon JI (1987). Expression of rat intestinal fatty acid-binding protein in *Escherichia coli*. Purification and comparison of ligand binding characteristics with that *of Escherichia coli*-derived rat liver fatty acid-binding protein. Journal of Biological Chemistry.

[ref-34] Lu L, Cao H, He S, Wei R, Diong M (2011). *Bacillus amyloliquefaciens* G1: a potential antagonistic bacterium against eel-pathogenic *Aeromonas hydrophila*. Evidence-Based Complementary and Alternative Medicine.

[ref-35] Mallo JJ, Gracia MI, Honrubia P, Sedano G (2010). Use of a *Bacillus amyloliquefaciens* probiotic in broiler farms. Poultry Science.

[ref-36] Martínez-Barberá JP, Pendón C, Martí-Palanca H, Calduch-Giner JA, Rodríguez RB, Valdivia MM, Pérez-Sánchez J (1995). The use of recombinant gilthead sea bream (*Sparus aurata*) growth hormone for radioiodination and standard preparation in radioimmunoassay. Comparative Biochemistry and Physiology Part A: Physiology.

[ref-37] Miller DW, Prosser Z, Chee EYW, Hansen CF, Dunshea FR, Mullan BP, Pluske JR (2016). Dietary stimulation of the endogenous somatotropic axis in weaner and grower-finisher pigs using medium chain triglycerides and cysteamine hydrochloride. Journal of Animal Science and Biotechnology.

[ref-38] Mulero I, Sepulcre MP, Meseguer J, García-Ayala A, Mulero V (2007). Histamine is stored in mast cells of most evolutionarily advanced fish and regulates the fish inflammatory response. Proceedings of the National Academy of Sciences of the United States of America.

[ref-39] Ng WK, Koh CB (2016). The utilization and mode of action of organic acids in the feeds of cultured aquatic animals. Reviews in Aquaculture.

[ref-40] Nordrum S, Olli JJ, Røsjø C, Holm H, Krogdahl Å (2003). Effects of graded levels of medium chain triglycerides and cysteine on growth, digestive processes and nutrient utilization in sea water reared Atlantic salmon (*Salmo salar*, L.) under ad libitum feeding regime. Aquaculture Nutrition.

[ref-41] Ooyama K, Kojima K, Aoyama T, Takeuchi H (2009). Decrease of food intake in rats after ingestion of medium-chain triacylglycerol. Journal of Nutritional Science and Vitaminology.

[ref-42] Palenzuela O, Sitjà-Bobadilla A, Álvarez Pellitero P (1996). Isolation and partial characterization of serum immunoglobulins from sea bass (*Dicentrarchus labrax* L.) and gilthead sea bream (*Sparus aurata* L.). Fish & Shellfish Immunology.

[ref-43] Pérez-Sánchez J, Benedito-Palos L, Estensoro I, Petropoulos Y, Calduch-Giner JA, Browdy CL, Sitjà-Bobadilla A (2015). Effects of dietary NEXT ENHANCE^®^150 on growth performance and expression of immune and intestinal integrity related genes in gilthead sea bream (*Sparus aurata* L.). Fish & Shellfish Immunology.

[ref-44] Pérez-Sánchez J, Estensoro I, Redondo MJ, Calduch-Giner JA, Kaushik S, Sitjà-Bobadilla A (2013). Mucins as diagnostic and prognostic biomarkers in a fish-parasite model: transcriptional and functional analysis. PLOS ONE.

[ref-45] Piazzon MC, Galindo-Villegas J, Pereiro P, Estensoro I, Calduch-Giner JA, Gomez-Casado E, Novoa B, Mulero V, Sitjà-Bobadilla A, Pérez-Sánchez J (2016). Differential modulation of IgT and IgM upon parasitic, bacterial, viral, and dietary challenges in a Perciform fish. Frontiers in Immunology.

[ref-46] Raida MK, Larsen JL, Nielsen ME, Buchmann K (2003). Enhanced resistance of rainbow trout, *Oncorhynchus mykiss* (Walbaum), against *Yersinia ruckeri* challenge following oral administration of *Bacillus subtilis* and *B. licheniformis* (BioPlus2B). Journal of Fish Diseases.

[ref-47] Ran C, Carrias A, Williams MA, Capps N, Dan BCT, Newton JC, Kloepper JW, Ooi EL, Browdy CL, Terhune JS, Liles MR (2012). Identification of *Bacillus* strains for biological control of catfish pathogens. PLOS ONE.

[ref-48] Reite OB, Evensen O (2006). Inflammatory cells of teleostean fish: a review focusing on mast cells/eosinophilic granule cells and rodlet cells. Fish & Shellfish Immunology.

[ref-49] Ridha MT, Azad IS (2012). Preliminary evaluation of growth performance and immune response of Nile tilapia *Oreochromis niloticus* supplemented with two putative probiotic bacteria. Aquaculture Research.

[ref-50] Rubin D, Helwig U, Pfeuffer M, Auinger A, Ruether A, Matusch D, Darabaneanu S, Freitag-Wolf S, Nothnagel M, Schreiber S, Schrezenmeir J (2012). The effect of FABP2 promoter haplotype on response to a diet with medium-chain triacylglycerols. Genes and Nutrition.

[ref-51] Saera-Vila A, Benedito-Palos L, Sitjà-Bobadilla A, Nácher-Mestre J, Serrano R, Kaushik S, Pérez-Sánchez J (2009). Assessment of the health and antioxidant trade-off in gilthead sea bream (*Sparus aurata* L.) fed alternative diets with low levels of contaminants. Aquaculture.

[ref-52] Sánchez J, Quiles A, Espinel AE, Díaz D, Gracia MI (2006). Effect of supplementing a probiotic feed additive on performance and digestibility of broilers. Poultry Science.

[ref-53] Scharrer E, Langhans W (1986). Control of food intake by fatty acid oxidation. American Journal of Physiology–Regulatory Integrative and Comparative Physiology.

[ref-54] Seal BS, Lillehoj HS, Donovan DM, Gay CG (2013). Alternatives to antibiotics: a symposium on the challenges and solutions for animal production. Animal Health Research Reviews/Conference of Research Workers in Animal Diseases.

[ref-55] Segner H, Sundh H, Buchmann K, Douxfils J, Sundell KS, Mathieu C, Ruane N, Jutfelt F, Toften H, Vaughan L (2012). Health of farmed fish: its relation to fish welfare and its utility as welfare indicator. Fish Physiology and Biochemistry.

[ref-56] Selim KM, Reda RM (2015). Improvement of immunity and disease resistance in the Nile tilapia, *Oreochromis niloticus*, by dietary supplementation with *Bacillus amyloliquefaciens*. Fish and Shellfish Immunology.

[ref-57] Sheng YH, Lourie R, Lindén SK, Jeffery PL, Roche D, Tran TV, Png CW, Waterhouse N, Sutton P, Florin THJ, McGuckin MA (2011). The MUC13 cell—surface mucin protects against intestinal inflammation by inhibiting epithelial cell apoptosis. Gut.

[ref-58] Sheng YH, Triyana S, Wang R, Das I, Gerloff K, Florin TH, Sutton P, McGuckin MA (2013). MUC1 and MUC13 differentially regulate epithelial inflammation in response to inflammatory and infectious stimuli. Mucosal Immunology.

[ref-59] Silva TFA, Petrillo TR, Yunis-Aguinaga J, Marcusso PF, Claudiano GS, Moraes FR, Moraes JRE (2015). Effects of the probiotic *Bacillus amyloliquefaciens* on growth performance, hematology and intestinal morphometry in cage-reared Nile tilapia. Latin American Journal of Aquatic Research.

[ref-60] Simó-Mirabet P, Bermejo-Nogales A, Calduch-Giner JA, Pérez-Sánchez J (2017). Tissue-specific gene expression and fasting regulation of sirtuin family in gilthead sea bream (*Sparus aurata*). Journal of Comparative Physiology B: Biochemical, Systemic, and Environmental Physiology.

[ref-61] Sitjà-Bobadilla A, Peña Llopis S, Gómez-Requeni P, Médale F, Kaushik S, Pérez-Sánchez J (2005). Effect of fish meal replacement by plant protein sources on non-specific defense mechanisms and oxidative stress in gilthead sea bream (*Sparus aurata*). Aquaculture.

[ref-62] Skřivanová E, Molatová Z, Skřivanová V, Marounek M (2009). Inhibitory activity of rabbit milk and medium-chain fatty acids against enteropathogenic *Escherichia coli* O128. Veterinary Microbiology.

[ref-63] Sturn A, Quackenbush J, Trajanoski Z (2002). Genesis: cluster analysis of microarray data. Bioinformatics.

[ref-64] Suiryanrayna MVAN, Ramana JV (2015). A review of the effects of dietary organic acids fed to swine. Journal of Animal Science and Biotechnology.

[ref-65] Sundell KS, Rønnestad I (2011). Integrated function and control of the gut— Intestinal absorption. Encyclopedia of Fish Physiology.

[ref-66] Trushenski JT (2009). Saturated lipid sources in feeds for sunshine bass: Alterations in production performance and tissue fatty acid composition. North American Journal of Aquaculture.

[ref-67] Vargas RJ, Dotta G, Mouriño JL, Silva BC, Fracalossi DM (2013). Dietary lipid sources affect freshwater catfish jundiá, *Rhamdia quelen*, survival, when challenged with *Aeromonas hydrophila*. Acta Scientiarum. Animal Sciences.

[ref-68] Vega-Rubín de Celis S, Rojas P, Gómez-Requeni P, Albalat A, Gutiérrez J, Médale F, Kaushik SJ, Navarro I, Pérez-Sánchez J (2004). Nutritional assessment of somatolactin function in gilthead sea bream (*Sparus aurata*): concurrent changes in somatotropic axis and pancreatic hormones. Comparative Biochemistry and Physiology. Part A, Molecular & Integrative Physiology.

[ref-69] Wang J, Wu X, Simonavicius N, Tian H, Ling L (2006). Medium-chain fatty acids as ligands for orphan G protein-coupled receptor GPR84. Journal of Biological Chemistry.

[ref-70] Wang YB, Li JR, Lin J (2008). Probiotics in aquaculture: challenges and outlook. Aquaculture.

[ref-71] Williams I, Williams KC, Smith DM, Jones M (2006). Polka-dot grouper, *Cromileptes altivelis*, can utilize dietary fat efficiently. Aquaculture Nutrition.

[ref-72] Zhang H, Li Y, Hou X, Zhang L, Wang T (2016). Medium-chain TAG improve energy metabolism and mitochondrial biogenesis in the liver of intra-uterine growth-retarded and normal-birth-weight weanling piglets. The British Journal of Nutrition.

[ref-73] Zhang YA, Salinas I, Li J, Parra D, Bjork S, Xu Z, LaPatra SE, Bartholomew J, Sunyer JO (2010). IgT, a primitive immunoglobulin class specialized in mucosal immunity. Nature Immunology.

[ref-74] Zorriehzahra MJ, Delshad ST, Adel M, Tiwari R, Karthik K, Dhama K, Lazado CC (2016). Probiotics as beneficial microbes in aquaculture: an update on their multiple modes of action: a review. Veterinary Quarterly.

