# Supplementary material for: Sodium salt medium-chain fatty acids and Bacillus-based probiotic strategies to improve growth and intestinal health of gilthead sea bream (Sparus aurata)
Source: PeerJ. 2017 Dec 4;5:e4001. doi: 10.7717/peerj.4001 (PMC5719961; doi:10.7717/peerj.4001)
Supplement: Table S1 [file peerj-05-4001-s001.docx]

**Supplementary Table 1 –** Forward and reverse primers used for real-time qPCR.

| Gene name |  | Symbol |  | Primer sequence |  |
| --- | --- | --- | --- | --- | --- |
| Sirtuin1 |  | *sirt1* | F | GGT TCC TAC AGT TTC ATC CAG CAG CAC ATC |  |
|  |  |  | R | CCT CAG AAT GGT CCT CGG ATC GGT CTC |  |
|  |  |  |  |  | |
| Sirtuin2 |  | *sirt2* | F | GAA CAA TCC GAC GAC AGC AGT GAA G |  |
|  |  |  | R | AGG TTA CGC AGG AAG TCC ATC TCT |  |
|  |  |  |  |  | |
| Sirtuin3 |  | *sirt3* | F | CTG CCA AGT CCT CAT CCC |  |
|  |  |  | R | CTT CAC CAG ACG AGC CAC |  |
|  |  |  |  |  | |
| Sirtuin4 |  | *sirt4* | F | GGC TGG CGG AGT CGG ATG |  |
|  |  |  | R | TCC TGA ATA CAC CTG TGA CGA AGA C |  |
|  |  |  |  |  | |
| Sirtuin5 |  | *sirt5* | F | CAG ACA TCC TAA CCC GAG CAG AG |  |
|  |  |  | R | CCA CGA GGC AGA GGT CAC A |  |
|  |  |  |  |  | |
| Sirtuin6 |  | *sirt6* | F | ACT CCA CCA CCA CCG ATG TCA A |  |
|  |  |  | R | CTC CTC CTC CTT CAC CTT TCG CTT TG |  |
|  |  |  |  |  | |
| Sirtuin7 |  | *sirt7* | F | CTG GAG CAA CCT CTA AAC TGG AA |  |
|  |  |  | R | CAC CTT CAG ACT GGA GCC TAA |  |
|  |  |  |  |  | |
| Occludin |  | *ocln* | F | GTG TCA GAA CCT CTA CCA GAC CAG CTA CTC |  |
|  |  |  | R | GAA AGC CTC CCA CTC CTC CCA TCT |  |
|  |  |  |  |  |  |
| Claudin-12 |  | *cldn12* | F | CTC TCA GGG CTA CAC ATC TAC CTA TGC |  |
|  |  |  | R | ACA TTC GTG AGC GGC TGG AG |  |
|  |  |  |  |  |  |
| Claudin-15 |  | *cldn15* | F | CCG ATT GTG GAA GTA GTG GCT CTG GT |  |
|  |  |  | R | CAG CAT CAC CCA ACC GAC GAA CC |  |
|  |  |  |  |  |  |
| Cadherin-1 |  | *cdh1* | F | TGC TCC ATA CAG CGT CAC CTT ACA |  |
|  |  |  | R | CTC GTT CAT CCT AGC CGT CCA GTT |  |
|  |  |  |  |  |  |
| Cadherin-17 |  | *cdh17* | F | GAT GCC CGC AAC CCA GAG |  |
|  |  |  | R | CCG TTG ATT CAC TGC CGT AGA C |  |
|  |  |  |  |  |  |
| Intestinal-type alkaline phosphatase |  | *alpi* | F | CCG CTA TGA GTT GGA CCG TGA T |  |
|  |  |  | R | GCT TTC TCC ACC ATC TCA GTA AGG G |  |
|  |  |  |  |  |  |
| Liver type fatty acid-binding protein |  | *fabp1* | F | GTC CTC GTC AAC ACC TTC ACC AT |  |
|  |  |  | R | CGC CTT CAT CTT CTC GCC AGT |  |
|  |  |  |  |  |  |
|  |  |  |  |  |  |
| Intestinal fatty acid-binding protein |  | *fabp2* | F | CGA GCA CAT TCC GCA CCA AAG |  |
|  |  |  | R | CCC ACG CAC CCG AGA CTT C |  |
|  |  |  |  |  |  |
| Ileal fatty acid-binding protein |  | *fabp6* | F | ACC CAG GAC GGC AAT ACC |  |
|  |  |  | R | CGA CGG TGA AGT TGT TGG T |  |
|  |  |  |  |  |  |
| Mucin 2 |  | *muc2* | F | ACG CTT CAG CAA TCG CAC CAT |  |
|  |  |  | R | CCA CAA CCA CAC TCC TCC ACA T |  |
|  |  |  |  |  |  |
| Mucin 13 |  | *muc13* | F | TTC AAA CCC GTG TGG TCC AG |  |
|  |  |  | R | GCA CAA GCA GAC ATA GTT CGG ATA T |  |
|  |  |  |  |  |  |
| Intestinal mucin |  | *i-muc* | F | GTG TGA CCT CTT CCG TTA |  |
|  |  |  | R | GCA ATG ACA GCA ATG ACA |  |
|  |  |  |  |  |  |
| Transcription factor HES-1-B |  | *hes1-b* | F | GCC TGC CGA TAT GAT GGA A |  |
|  |  |  | R | GGA GTT GTG TTC ATG CTT GC |  |
|  |  |  |  |  |  |
| Krueppel-like factor 4 |  | *klf4* | F | ACA TCA CCG CAC GCA CAC |  |
|  |  |  | R | AAC CAC AGC CCT CCC AGT C |  |
|  |  |  |  |  |  |
| Tumor necrosis factor-alpha |  | *tnfα* | F | CAG GCG TCG TTC AGA GTC TC |  |
|  |  |  | R | CTG TGG CTG AGA GCT GTG AG |  |
|  |  |  |  |  |  |
| Interleukin-1 beta |  | *il1β* | F | GCG ACC TAC CTG CCA CCT ACA CC |  |
|  |  |  | R | TCG TCC ACC GCC TCC AGA TGC |  |
|  |  |  |  |  |  |
| Interleukin-6 |  | *il6* | F | TCT TGA AGG TGG TGC TGG AAG TG |  |
|  |  |  | R | AAG GAC AAT CTG CTG GAA GTG AGG |  |
|  |  |  |  |  |  |
| Interleukin-8 |  | *il8* | F | CAG CAG AGT CTT CAT CGT CAC TAT TG |  |
|  |  |  | R | AGG CTC GCT TCA CTG ATG G |  |
|  |  |  |  |  |  |
| Interleukin-10 |  | *il10* | F | AAC ATC CTG GGC TTC TAT CTG |  |
|  |  |  | R | GTG TCC TCC GTC TCA TCT G |  |
|  |  |  |  |  |  |
| CD4 |  | *cd4* | F | TCC TCC TCC TCG TCC TCG TT |  |
|  |  |  | R | GGTGTCTCATCTTCCGCTGTCT |  |
|  |  |  |  |  |  |
| CD8 alpha |  | *cd8α* | F | GCA GCA ACG GTA ACA CGA ACG |  |
|  |  |  | R | CCAGTATGAGCGGAGTACAGAACA |  |
|  |  |  |  |  |  |
| CD8 beta |  | *cd8β* | F | CCG AAA TGT GGA AGA CTG GAA CTC |  |
|  |  |  | R | CTTTGGAGGTAAGGTTGGAGGGAT |  |
|  |  |  |  |  |  |
| Galectin-1 |  | *lgals1* | F | GTG TGA GGA GGT CCG TGA TG |  |
|  |  |  | R | ACT GTA GAG CCG TCC GAT AGG |  |
| Galectin-8 |  | *lgals8* | F | GGC GGT GAA CGG CGG TCA |  |
|  |  |  | R | GCT CCA GCT CCA GTC TGT GTT GAT AC |  |
|  |  |  |  |  |  |
| Secreted Immunoglobulin M |  | *sIgM* | F | ACC TCA GCG TCC TTC AGT GTT TAT GAT GCC |  |
|  |  |  | R | CAG CGT CGT CGT CAA CAA GCC AAG C |  |
|  |  |  |  |  |  |
| Secreted Immunoglobulin T |  | *sIgT* | F | GCT GTC AAG GTG GCC CCA AAA G |  |
|  |  |  | R | CAA CAT TCA TGC GAG TTA CCC TTG GC |  |
|  |  |  |  |  |  |
| Membrane Immunoglobulin M |  | *mIgM* | F | GCTATGGAGGCGGAGGAAGATAACA |  |
|  |  |  | R | GCAGAGTGATGAGGAAGAGAAGGATGAA |  |
|  |  |  |  |  |  |
| Membrane Immunoglobulin T |  | *mIgT* | F | AGA CGA TGC CAG TGA AGA GGA TGA GT |  |
|  |  |  | R | CGA AGG AGG AGG CTG TGG ACC A |  |
|  |  |  |  |  |  |
| β-Actin |  | *actb* | F | TCCTGCGGAATCCATGAGA |  |
|  |  |  | R | GACGTCGCACTTCATGATGCT |  |
