# Supplementary material for: Sodium salt medium-chain fatty acids and Bacillus-based probiotic strategies to improve growth and intestinal health of gilthead sea bream (Sparus aurata)
Source: PeerJ. 2017 Dec 4;5:e4001. doi: 10.7717/peerj.4001 (PMC5719961; doi:10.7717/peerj.4001)
Supplement: Data S1 [file peerj-05-4001-s002.pdf]

Growth Performance

DIET	TANK	IBW	FBW	SGR	FE	INTAKE G/FISH	WEIGHT GAIN
D1	T21	28.41			0.835	155.40	
D1	T32	29.83	148.15	1.60	0.808	146.35	396.64
D1	T24	29.23	147.17	1.62	0.818	144.20	403.50
D2	T31	29.75	170.25	1.74	0.828	169.75	472.27
D2	T23	29.3	163.18	1.72	0.843	158.88	456.94
D2	T34	29.39	169.64	1.75	0.869	161.45	477.21
D3	T22	29.73	143.43	1.57	0.830	139.80	382.44
D3	T35	28.88	149.40	1.64	0.844	142.74	417.33
D3	T36	31.85	144.65	1.51	0.832	137.16	354.17

Biometric Indexes

DIET	FISH	WEIGHT	LENGHT	Hc	Hb	LIVER	Viscera	FAT	INT. WEIGHT	INT. LENGHT	HSI	VSI	MSI	II-I	GI	CF
D1	21, 1	188	18.5	44		3.29	13.27	0.9			1.75	7.06	0.48			2.97
D1	21, 2	172	18.5	40	10.7	2.13	12.5	2.96			1.24	7.27	1.72			2.72
D1	21, 3	141	18.7	35	10.8	2.5	8.9	1.6			1.77	6.31	1.13			2.16
D1	21, 4	163	18.3		10.9	2.65	11.42	3.09	4.95	10.5	1.63	7.01	1.90	14.08		2.66
D1	21, 5	173	17.5		7.6	2.63	11.34	1.29	3.41	13.7	1.52	6.55	0.75	6.73	0.13	3.23
D1	21, 6	149	18.2	44	9.5	3	9.1	0.8	2.1	13	2.01	6.11	0.54	6.78	0.10	2.47
D1	24, 1	170	18	40	10.4	2.91	8.73	1.09			1.71	5.14	0.64			2.91
D1	24, 2	161	18.1	40	9.3	3.83	10.32	1.15			2.38	6.41	0.71			2.72
D1	24, 3	191	17.9	40	10.4	3.2	12	1.8			1.68	6.28	0.94			3.33
D1	24, 4	146	17.3	46	10.5	2.52	9.49	3.12	2.43	9	1.73	6.50	2.14	20.03	0.33	2.82
D1	24, 5	158	17.8	40	9.4	2.88	12.83	3.25	1.99	12.1	1.82	8.12	2.06	8.92	0.11	2.80
D1	24, 6	143.5	16.8	39	9.9	2.6	9.4	1.5	1.6		1.81	6.55	1.05			3.03
D1	32, 1	157	17	42	10.2	2.38	9.71	1.75			1.52	6.18	1.11			3.20
D1	32, 2	172	18.5	55	11.8	3.4	10.29	0.61			1.98	5.98	0.35			2.72
D1	32, 3	143	16.57	54	11.8	2.7	10	1.7			1.89	6.99	1.19			3.14
D1	32, 4	137	17.4	42	10.9	2.1	9.91	3.01	2.9	11	1.53	7.23	2.20	10.29	0.22	2.60
D1	32, 5	173	18.3	47	10.6	2.72	12.49	2.6	1.96	12.2	1.57	7.22	1.50	9.53	0.11	2.82
D1	32, 6	118	16.1	48	9.8	2	7.5	1.1	2.5	6.7	1.69	6.36	0.93			2.83
D2	23, 1	176.5	18	35	12.3	3.04	9.85	1.49			1.72	5.58	0.84			3.03
D2	23, 2	220.5	20.3	34	10.8	3.38	13.39	2.32			1.53	6.07	1.05			2.64
D2	23, 3	188	18.5	45	6.4	4.2	14.4	2.7			2.23	7.66	1.44			2.97
D2	23, 4	175	17.5	51	11.9	4.18	12.23	3.21	2.41	10	2.39	6.99	1.83	17.50	0.24	3.27
D2	23, 5	174	18.5	49	11.5	3.76	10.73	1.48	2.63	10.3	2.16	6.17	0.85	15.92	0.24	2.75
D2	23, 6	139	16.7	45	11.6	3.1	10.7	2.9	2.3	8.5	2.23	7.70	2.09	22.63	0.37	2.98
D2	31, 1	142	16.5	40	9.8	2.61	8.23	2.19			1.84	5.80	1.54			3.16
D2	31, 2	148	17	40	9.9	3.05	13.69	3.65			2.06	9.25	2.47			3.01
D2	31, 3	169	17.8	34	8.8	3.2	9.2	0.7			1.89	5.44	0.41			3.00
D2	31, 4	167	18	37	10.3	4.38	12.3	1.89	3.46	10.6	2.62	7.37	1.13	14.02	0.29	2.86
D2	31, 5	189	18.1	30	8.7	4.47	15.12	3.81	3.19	12.6	2.37	8.00	2.02	9.45	0.16	3.19
D2	31, 6	193.5	16.8	35	10.6	4.4	13.8	1.2	1.8	9.6	2.27	7.13	0.62	21.87	0.20	4.08
D2	34, 1	183	18.5	32	8.9	3.5	14.7	4.06			1.91	8.03	2.22			2.89
D2	34, 2	159	18.3	37	9.9	3.14	11.47	2.19			1.97	7.21	1.38			2.59
D2	34, 3	162	17.3	45	10.6	3.3	12.1	1.9			2.04	7.47	1.17			3.13
D2	34, 4	184	19.3	34	9.4	3.1	12.35	3.73	2.95	11.3	1.68	6.71	2.03	12.75	0.21	2.56
D2	34, 5	152	17.8	43	11.1	3	10.15	1.55	1.78	8.5	1.97	6.68	1.02	24.75	0.29	2.70
D2	34, 6	193.5	18.6	46	12.2	3.4	10.2	0.6	2.9	8.6	1.76	5.27	0.31	30.42	0.46	3.01
D3	22, 1	161.5	17.5	41	9.9	2.29	8.22	1.13			1.42	5.09	0.70			3.01
D3	22, 2	186.5	19	34	9.1	3.87	8.9	1.93			2.08	4.77	1.03			2.72
D3	22, 4	150	17.5		9.3	2.84	11.43	2.94	3.28	15.1	1.89	7.62	1.96	4.03	0.09	2.80
D3	22, 5	129.5	17.3	46	10.7	2.41	7.65	1.03	2.92	10.1	1.86	5.91	0.80	12.57	0.28	2.50

D3	22, 6	169	18.6		8.1	2.9	9.3	0.9	2.2	9.2	1.72	5.50	0.53	21.70	0.28	2.63
D3	35, 1	126	15	42	10.3	2.37	8.9	1.39			1.88	7.06	1.10			3.73
D3	35, 2	160	17.7	39	9.2	3.57	10.86	1.25			2.23	6.79	0.78			2.89
D3	35, 3	161	17.3	56	11.1	3.6	10.9	1.3			2.24	6.77	0.81			3.11
D3	35, 4	163	18.7	36	8.7	3.19	11.43	2.4	2.87	8.5	1.96	7.01	1.47	26.54	0.47	2.49
D3	35, 5	140	17.1	40	8.7	2.45	12.51	3.73	1.74	12.2	1.75	8.94		7.71	0.10	2.80
D3	35, 6	150	17.1	46	11.1	3.3	11	2.2	1.9	13.2	2.20	7.33	1.47	6.52	0.08	3.00
D3	36, 1	123	16.4	33	10	1.77	7.77	1.37			1.44	6.32	1.11			2.79
D3	36, 2	141	17.3	35	10.9	2.27	10.19	1.67			1.61	7.23	1.18			2.72
D3	36, 3	147	16.9	30	8	2.3	11.1	2.9			1.56	7.55	1.97			3.05
D3	36, 4	170	17.7	32	9.9	2.25	10.63	2.55	2.97	13.5	1.32	6.25	1.50	6.91	0.12	3.07
D3	36, 5	133	17.2	38	10.4	1.93	10.22	2.51	1.37	8.8	1.45	7.68	1.89	19.52	0.20	2.61
D3	36, 6	153	17.3	30	7.9	2.4	11.4	6.4	1.6	14.2	1.57	7.45		5.34	0.06	2.95

Biochemistry

DIET	FISH	Glucose (mg/dl)	Protein (ug/ml)	Protein g/l	Cholest. tot (ug/poc.)	Cholesterol (mg/dl)	TG (ug/well)	Triglicerides (mM)	Raw data antioxidant	Antioxidant act(trolox) mM	ortisol ng/ml	IGF-I ng/ml	GH (ng/ml)	ative complement	IgM	Lysozyme
D1	21.1		15.77	39.425	7.15	178.75	54.22	12.15	0.082	0.82	3.71		10.192	36.76	1.4226	0
	21.2	40.54	18.47	46.175	8.27	206.75	17.97	4.03	0.086	0.86	6.75	88.368	18.384	22.27	1.29835	450
	21.3	39.07	16.09	40.225	6.53	163.25	37.49	8.40	0.093	0.93	7.42	72.576	8.552	17.12	1.5451	133.33
	24.1	29.5	15.91	39.775	10.42	260.5	60.56	13.57	0.098	0.98			17.728	6.25	1.04065	70
	24.2	39.8	15.01	37.525	6.67	166.75	12.77	2.86	0.085	0.85	10.31	90.048	10.904	38.99	1.29405	92.2
	24.3	33.8	14.81	37.025	7.58	189.5	22.64	5.07	0.09	0.90		77.112	8.752	45.25	1.33045	28.89
	32.1	35.9	14.85	37.125	7.68	192	14.68	3.29	0.092	0.92	8.56	90.048	9.804	46.40	1.04935	0
	32.2	35.8	17.94	44.85	6.69	167.25	37.71	8.45	0.096	0.96	10.40	84.336	7.66	7.73	1.3846	12.2
	32.3	40.6	17.12	42.8	6.87	171.75	21.06	4.72	0.091	0.91	10.79		10.448	12.16	1.08945	56.65
D2	23.1	47.2	17.78	44.45	6.71	167.75	11.09	2.48	0.1	1.00			8.692	31.25	0.76415	0
	23.2	47.9	15.08	37.7	8.39	209.75	7.94	1.78	0.098	0.98	7.70			29.54	1.09155	0
	23.3	47.8	17.93	44.825	8.71	217.75	11.71	2.62	0.095	0.95	6.06	82.656		78.43	0.9018	75.5555
	31.1	37.2	13.58	33.95	7.52	188	44.43	9.95	0.7			83.664	12.768	44.44	1.55445	0
	31.2	55.8	15.92	39.8	8.33	208.25	34.58	7.75	0.088	0.88	4.64	115.92	8.784	2.13	1.0648	56.6666
	31.3	38.9	16.9	42.25	7.25	181.25	15.31	3.43	0.081	0.81	5.56	110.88	11.928	1.87	1.48245	36.67
	34.1	34.7	15.18	37.95	6.67	166.75	10.34	2.32	0.078	0.78	6.55	71.4	8.544	0.38	1.06185	0
	34.2	43.3	17.4	43.5	8.27	206.75	19.57	4.38	0.086	0.86	9.94	116.76	10.536	0.14	0.89175	0
	34.3	40.1	15.4	38.5	7.11	177.75	34.91	7.82	0.079	0.79	7.98	80.136	15.02	32.68	1.2447	93.333
D3	22.1	36.4	16.63	41.575	7.26	181.5	12.42	2.78	0.087	0.87	10.5	27.048	17.908	26.56	1.04765	0
	22.2	41.1	15.12	37.8	7.24	181	17.63	3.95	0.082	0.82	6.19	103.992	19.188	23.95	0.5399	0
	22.3	44.6	18.19	45.475	7.72	193	10.37	2.32	0.092	0.92		87.024	12.16	55.71	1.18375	18.89
	35.1	43.5	15.49	38.725	7.11	177.75	35.63	7.98	0.085	0.85	4.32	102.984	11.552	19.82	1.47465	15.5556
	35.2	35.1	14.68	36.7	6.1	152.5	15.15	3.39	0.082	0.82	0.91	97.44	7.828	28.86	0.8134	1.111
	35.3	48.5	15.25	38.125	7.27	181.75	45.7		0.088	0.88	1.9	93.912	12.216	17.56	1.8599	0
	36.1	37.1	16.02	40.05	6.93	173.25	15.59	3.49	0.11		5.2	77.28	15	47.51	0.81545	67.66
	36.2	43.6	12.15	30.375	5.4	135	9.98	2.24	0.089		4.97	83.328	9.86	39.14	0.76085	17.7777
	36.3	52.7	16.72	41.8	6.15	153.75	7.33	1.64	0.12		3.92	72.072	18.776	2.94	0.86155	0

Gene Expression - AI D1

	21.1	24.1	32.1	21.2	24.2	32.2	21.3	21.3	32.3
	Expression	Expression	Expression	Expression	Expression	Expression	Expression	Expression	Expression
OCLN	5.776	7.362	8.754	3.272	7.413		5.776	6.148	
CLDN12	0.707	0.953	1.206	0.543	1.087		0.790	1.266	
CLDN15	26.723	19.698	38.055	19.293	27.096		17.630	42.814	
CDH1	17.630	17.268	26.538		16.912		25.107	33.591	
CDH17	56.103	79.341	77.708	51.984	83.865		49.522	105.420	
ALPI		97.681	92.411	83.865	138.141		133.436	130.690	
FABP1	108.383	147.033	176.069	95.670	207.937		192.672	188.706	
FABP2	168.897		266.871	170.072	608.874		265.028	704.277	
FABP6	0.014	0.022	0.008	0.007			0.013	0.076	
MUC2	38.586	36.758	53.076	30.484	41.933		36.504	53.446	
MUC13	120.259	116.970	167.730	92.411	135.298		196.720	215.269	
I-MUC	0.198	0.112	0.131	0.187	0.150		0.121	0.054	
HES1-B	3.340	5.278	2.158	2.071	2.770		4.170	6.916	
KLF4	1.972	3.811	3.031	2.000	2.969		3.074	3.294	
TNF-α	0.144	0.188	0.107	0.039	0.157		0.123	0.171	
IL-1β	0.051	0.108	0.083	0.050	0.090		0.092	0.071	
IL-6	0.016	0.025	0.031	0.011	0.013		0.063	0.026	
IL-8	0.140	0.323		0.122	0.283		0.275	0.330	
IL-10	0.062	0.093	0.164	0.087	0.105		0.143		
CD4-full	0.403	0.441	0.590	0.272	0.325		0.480	1.214	
CD8a	0.473	0.847	0.933	0.514	0.871		0.801	1.181	
CD8b	0.063	0.132	0.177	0.060	0.116		0.100	0.156	
LGALS1	4.347	5.736	6.821	2.585	7.781		5.169	7.413	
LGALS8	3.630	2.603	4.563	2.462	4.500		3.317	5.278	
IgM	2.313	1.580	16.912	7.568	13.737		1.919	26.723	
IgT	0.041	0.064	0.135	0.085	0.069		0.066	0.243	
IgT-m	0.366	0.435	0.532	0.376	0.460		0.521		
mIgM	0.098	0.076	0.262	0.109	0.206		0.097	0.570	
SIRT1	0.629	1.028	1.223		0.933	1.214	0.993	1.376	0.807
SIRT2	0.824	1.050	1.357		1.580	1.385	1.102	1.569	0.871
SIRT3		0.420	0.697		0.337	0.674	0.366	0.774	0.374
SIRT4	0.111	0.136	0.233		0.139	0.133	0.154	0.228	0.070
SIRT5	0.790	1.007	1.310		1.223	0.993	0.959	1.636	0.737
SIRT6	0.147	0.259	0.349		0.235	0.248	0.261	0.316	0.125
SIRT7	0.183	0.222	0.285		0.277	0.323	0.232	0.354	0.193

Gene Expression - AI D2

	31.1	23.1	34.1	31.2	23.2	34.2	31.3	31.3	34.3
	Expression	Expression	Expression	Expression	Expression	Expression	Expression	Expression	Expression
OCLN	5.098	6.021	6.821	4.993	5.618	6.498	6.635	6.635	5.897
CLDN12	1.223	1.157	0.829	1.007	1.125	0.829	1.028	1.231	1.042
CLDN15	25.813	20.535	26.173	23.264	20.112	25.107	21.706	23.588	25.281
CDH1	17.509	13.642	12.996	17.509	12.210	14.825	14.123	15.137	12.042
CDH17	53.817	70.522	65.345	59.714	62.250	60.969	64.893	69.071	58.081
ALPI	137.187	135.298	95.010	162.017	102.537	123.640		103.968	135.298
FABP1	142.025	121.938	136.239	142.025	154.343	151.167	210.839	168.897	171.255
FABP2	265.028	1,045.516	487.751	474.413	948.826	714.109	1,278.290	666.287	729.114
FABP6	0.016	0.023	0.011	0.027	0.050	0.010	0.020		0.021
MUC2	46.851	55.330	56.886	38.055	37.792	53.817	32.900	41.070	36.504
MUC13	128.890	113.772	102.537	150.123	98.360	121.938	102.537	121.095	128.890
I-MUC	0.132	0.200	0.088	0.097	0.026	0.117	0.076	0.395	0.215
HES1-B	5.098	2.395	3.864	4.691	2.828	2.532	2.204	2.329	3.706
KLF4	3.580	3.117	3.053	2.585	1.828	2.657	2.346	2.532	2.428
TNF-α	0.128	0.093	0.094	0.176	0.103	0.070	0.133	0.075	0.129
IL-1β	0.094	0.065	0.072	0.084	0.060	0.071	0.103	0.077	0.101
IL-6	0.014	0.021		0.018	0.014	0.020	0.023	0.020	0.024
IL-8	0.146	0.123	0.147	0.171	0.072	0.129	0.105	0.114	0.184
IL-10	0.137	0.093	0.095	0.126	0.099	0.116	0.132	0.141	0.128
CD4-full	0.387	0.332	0.277	0.354	0.387	0.319	0.304	0.454	0.314
CD8a	1.366	0.883	0.518	0.871	1.102	0.547	0.979	0.693	0.763
CD8b	0.224	0.128	0.053	0.104	0.248	0.071	0.110	0.082	0.107
LGALS1	4.993	6.453	5.278	4.377	6.681	4.959	6.277	6.409	7.890
LGALS8	3.138	2.621	2.549	3.294	2.585	3.204	1.919	2.445	3.095
IgM	7.160	2.639	5.352	1.223	1.932	7.160	1.165	7.111	3.531
IgT	0.075	0.117	0.071	0.069	0.080	0.128	0.107	0.031	0.049
IgT-m	0.196	0.563	0.460	0.476	0.346	0.525	0.603	0.259	0.543
mIgM	0.186	0.125	0.118	0.120	0.111	0.148	0.060	0.207	0.240
SIRT1	1.057	0.717	0.841	0.871	0.633	0.908	0.883	0.859	0.753
SIRT2	1.149	1.231	1.050	1.057	0.993	1.021	0.940	0.966	1.064
SIRT3	0.314	0.293	0.406	0.289	0.349	0.363	0.328	0.354	0.429
SIRT4	0.210	0.129	0.130	0.137	0.110	0.122	0.098	0.105	0.136
SIRT5	1.283	0.946	1.117	0.847	1.079	0.835	1.173	0.993	1.050
SIRT6	0.250	0.212	0.210	0.192	0.166	0.209	0.219	0.213	0.171
SIRT7	0.295	0.250	0.245	0.232	0.200	0.253	0.237	0.206	0.255

Gene Expression - AI D3

	22.1	35.1	36.1	22.2	35.2	36.2	22.3	22.3	36.3
	Expression	Expression	Expression	Expression	Expression	Expression	Expression	Expression	Expression
OCLN	4.377	6.320	7.781	5.696	6.105	7.674	5.315	6.635	10.629
CLDN12	0.683	1.014	1.042	1.050	1.007	1.087	0.841	0.959	0.824
CLDN15	17.148	22.943	34.535	25.107	24.420	32.223	22.471	25.634	33.825
CDH1	11.959	15.032	18.896	15.889	17.388	12.729	12.641	15.242	14.026
CDH17	52.346	79.893	79.341	59.302	87.427	71.012	58.081	82.139	77.708
ALPI	71.506	89.884	135.298	105.420	97.006	123.640	59.302	116.970	80.449
FABP1	118.603	122.786	145.009	166.572	155.417	174.853	148.056	151.167	
FABP2	477.713	552.565	270.597	326.288	792.353	471.136	749.612	809.002	
FABP6	0.034	0.112	0.021	0.038	0.038	0.021	0.114	0.124	0.056
MUC2	28.246	35.753	42.224	42.814	34.060	36.758	43.411	54.948	33.359
MUC13	78.793	120.259	94.353	125.366	111.430	107.635	88.647	138.141	106.891
I-MUC	0.136	0.037	0.268	0.113	0.055	0.196	0.088	0.076	0.155
HES1-B	3.204	1.693	4.500	5.352	3.891	4.287	3.364	4.000	2.028
KLF4	1.828	3.031	3.294	3.972	1.972	3.387	3.387	4.438	3.010
TNF-α	0.113	0.093	0.146	0.110	0.143	0.159	0.118	0.112	0.109
IL-1β	0.060	0.050	0.110	0.143	0.070	0.090	0.076	0.098	0.080
IL-6	0.024	0.018	0.038	0.053	0.020	0.038	0.036		0.033
IL-8	0.119	0.125	0.382	0.154	0.182	0.180	0.135	0.139	0.124
IL-10	0.076	0.101	0.113	0.110	0.071	0.164	0.066	0.105	0.145
CD4-full	0.275	0.423	0.259	0.401	0.295	0.325	0.310	0.291	0.325
CD8a	0.678	0.637	0.889	0.812	0.435	0.732	0.521	0.859	0.865
CD8b	0.104	0.087	0.097	0.099	0.057	0.129	0.064	0.104	0.116
LGALS1	2.868	6.320	4.925	7.621	3.945	4.823	7.111	4.993	5.205
LGALS8	1.548	2.751	2.657	2.514	3.182	2.848	1.879	3.891	3.249
IgM	7.362	6.277	5.098	3.160	4.170	7.781		8.056	4.257
IgT	0.117	0.092	0.177	0.195	0.139	0.056	0.085	0.090	0.129
IgT-m	0.183	0.261	0.697	0.927	0.507	1.028	0.212	0.403	0.973
mIgM	0.098	0.117	0.128	0.178	0.063	0.198	0.096	0.150	0.154
SIRT1	0.559	0.747	0.865	0.801	0.707		0.702	0.812	0.927
SIRT2	0.678	0.959	0.914	0.933	0.933	1.223	0.707	1.165	0.824
SIRT3	0.206	0.356	0.384	0.395	0.301	0.454	0.262	0.384	0.328
SIRT4	0.087	0.139	0.128	0.130	0.157	0.133	0.083	0.154	0.079
SIRT5	0.435	1.007	1.283	0.946	0.835	1.414	0.722	0.933	1.064
SIRT6		0.224	0.210	0.192	0.210	0.210	0.140	0.180	0.202
SIRT7	0.132	0.204	0.207	0.177	0.177		0.131	0.213	0.225

Gene Expression - PI D1

	21.1	24.1	32.1	21.2	24.2	32.2	21.3	21.3	32.3
	Expression	Expression	Expression	Expression	Expression	Expression	Expression	Expression	Expression
OCLN	7.727	9.849	9.126	7.674	10.853		7.413		7.013
CLDN12	0.790	1.376	0.933	0.853	1.548		0.796		0.829
CLDN15	32.223	38.854	41.070	42.518	58.081		41.643		26.355
CDH1	10.056	13.833	11.314	8.754	15.242		16.795		9.514
CDH17	47.505	49.522	48.168	40.224	41.070		40.504		54.569
ALPI	42.814	30.274	22.162	15.889	18.896		35.261		36.252
FABP1	138.141	92.411	97.006	99.044	51.268		176.069		184.823
FABP2	657.114	439.586	504.951	487.751			556.408		995.999
FABP6	5,293.477	4,672.568	2,856.435	2,916.455	2,778.326		4,096.000		1,351.176
MUC2	33.825	39.671	50.914	25.813	39.397		45.255		30.910
MUC13	95.010	100.427	106.153	79.893	104.691		151.167		74.028
I-MUC	9.190	0.310	24.251	11.551	12.553		0.146	0.297	0.032
HES1-B	3.434	6.105	2.713	3.458	4.959		4.469		3.160
KLF4	1.866	2.250	1.972	1.828	3.138		1.959		2.462
TNF-α	0.091	0.158	0.136	0.093	0.097		0.139		0.110
IL-1β	0.099	0.159	0.079	0.113	0.130		0.127		0.081
IL-6	0.016	0.020	0.028	0.018	0.017		0.033		
IL-8	0.115	0.270	0.707	0.603	0.188		0.202		0.147
IL-10	0.110	0.207	0.164	0.172	0.134		0.156		0.156
CD4-full	0.460	0.785	0.559	0.467	0.551		0.551		0.500
CD8a	0.616	1.366	0.895	0.543			0.824		0.551
CD8b	0.080	0.212	0.150	0.142	0.178		0.107		0.095
LGALS1	8.456	13.086	8.340	10.126	14.520		8.056		8.938
LGALS8	2.329	3.681	2.532	3.053	4.595		2.266		2.514
IgM	2.378	27.096	17.509	4.959	22.471		2.949		5.938
IgT	0.019	0.038	0.136	0.093	0.032		0.040		0.022
IgT-m	0.521	0.847	0.688	0.511	0.369		0.473		0.688
mIgM	0.293	0.812	0.611	0.158	0.454		0.275		0.164
SIRT1	0.660	1.021	0.678	0.683	1.102		0.683	1.329	0.646
SIRT2	0.853	1.240	1.087	0.959		1.516	0.883	1.474	0.737
SIRT3	0.206	0.486	0.363	0.299	0.460	0.841	0.262	0.847	0.354
SIRT4	0.066	0.115	0.098	0.099	0.130	0.233	0.097	0.235	0.075
SIRT5	0.763	1.320	0.895	0.914	1.275	1.636	0.790	1.591	0.796
SIRT6	0.102	0.289	0.152	0.189	0.241	0.334	0.204		0.141
SIRT7	0.171	0.328	0.178	0.289	0.312	0.261	0.224	0.308	0.193

Gene Expression - PI D2

	31.1	23.1	34.1	31.2	23.2	34.2	31.3	31.3	34.3
	Expression	Expression	Expression	Expression	Expression	Expression	Expression	Expression	Expression
OCLN	10.483	10.339	9.000	15.348	10.703	12.210	6.727	9.190	8.056
CLDN12	1.310	1.257	0.908	1.828	1.647	1.717	0.859	1.275	0.835
CLDN15	49.180	45.255	33.825	75.061	67.649	77.708	19.293	29.651	40.504
CDH1	18.379	13.361	9.063	21.259	18.507	22.627	10.339	12.641	12.553
CDH17	41.070	62.683	75.061	40.504	49.867	38.055	48.840	69.551	51.625
ALPI	38.319	48.168	28.443	21.556	29.857	42.814	40.786	88.647	29.041
FABP1	97.006	205.074	155.417	3.630	81.572	21.112	184.823	261.379	219.793
FABP2	317.365	1,675.063	1,112.816		604.668		1,530.726	1,770.572	1,448.155
FABP6	3,104.188	2,628.456	4,576.408	1,530.726	4,039.609	1,438.152	1,332.574		1,858.599
MUC2	44.632	37.531	24.251	37.531	31.779	39.671	29.041	16.679	32.447
MUC13	191.341	113.772	103.968	124.500	140.070	127.116	85.627	107.635	101.125
I-MUC	17.753	0.441	0.141	132.514	61.393		0.210	0.046	0.143
HES1-B	4.563	2.621	4.959	8.938	4.408	6.320	1.741	3.630	4.287
KLF4	3.227	3.010	3.095	2.346	2.732	2.158	3.317	2.346	3.891
TNF-α	0.116	0.129	0.120	0.180	0.106	0.204	0.137	0.153	0.129
IL-1β	0.088	0.080	0.082	0.200	0.099	0.139	0.116	0.136	0.148
IL-6	0.031	0.012	0.026	0.020	0.014	0.035	0.015	0.017	0.038
IL-8	0.219	0.128	0.346	0.179	0.195	0.297	0.102	0.138	0.297
IL-10	0.218	0.138	0.173	0.131	0.127	0.252	0.125	0.125	0.170
CD4-full	0.529	0.532	0.483	0.620	0.500	0.946	0.392	0.285	0.351
CD8a	1.464	0.678	0.901	1.526	1.181	1.879	0.895	0.877	0.514
CD8b	0.264	0.137	0.108	0.233	0.196	0.346	0.129	0.150	0.088
LGALS1	9.918	9.849	11.632	9.254	11.551	15.242	6.727	8.000	11.713
LGALS8	4.347	3.364	2.676	7.568	5.938	6.964	1.636	2.868	2.676
IgM	12.641	9.646	4.595	5.464	3.138	29.243	1.580	2.378	4.084
IgT	0.043	0.099	0.032	0.032	0.124	0.213	0.112	0.060	0.029
IgT-m	0.261	0.712	1.283	0.973	0.865	1.537	0.753	0.441	0.620
mIgM	0.423	0.216	0.213	0.334	0.275		0.120	0.102	0.152
SIRT1	0.457	0.883	0.835	1.165	0.785	1.347	0.582	0.758	0.753
SIRT2	0.547	1.357	1.000	1.444	1.301	1.329	0.688	1.035	0.807
SIRT3	0.155	0.398	0.339	0.460	0.444	0.595	0.356	0.578	0.460
SIRT4	0.067	0.123	0.079	0.218	0.106	0.174	0.099	0.119	0.082
SIRT5	0.660	1.395	1.189	0.933	1.087	1.231	0.966	1.181	0.747
SIRT6	0.156	0.224	0.195	0.189	0.204	0.332	0.138	0.180	0.117
SIRT7	0.143	0.293	0.264	0.287	0.198	0.337	0.176	0.228	0.182

Gene Expression - PI D3

	22.1	35.1	36.1	22.2	35.2	36.2	22.13	22.13	36.3
	Expression	Expression	Expression	Expression	Expression	Expression	Expression	Expression	Expression
OCLN	7.568	6.681	11.794	17.753	8.340		5.938	8.515	11.081
CLDN12	0.895	0.877	1.310	1.972	1.347		0.908	0.946	1.007
CLDN15	32.000	23.588	64.445	103.968	41.933		28.641	49.522	45.570
CDH1	12.641	9.190	15.032		14.929		9.254	12.996	13.548
CDH17	45.887	60.548	54.192	30.274	44.324		49.522	46.527	68.594
ALPI	30.274	29.446	45.887	25.107	23.264		26.355	31.341	48.503
FABP1	173.645	159.786	103.968		46.851		165.421	104.691	242.191
FABP2	1,418.352	1,009.902	519.147		222.861		1,160.073	464.650	
FABP6	4,837.346	2,977.736	3,848.285		2,896.309		1,722.156	1,833.011	2,005.853
MUC2	25.992	26.723	41.070	55.330	43.713		32.672	54.569	35.753
MUC13	99.733	88.647	101.125	148.056	83.286		76.639	85.036	106.891
I-MUC	0.248	0.078	14.520	151.167	33.359		0.219	32.672	0.189
HES1-B	5.389	1.840	9.448	10.196	5.579		1.682	3.732	2.514
KLF4	3.053	2.282	3.758	2.189	3.249		2.114	2.928	3.364
TNF-α	0.136	0.101	0.166	0.237	0.222			0.118	0.152
IL-1β	0.087	0.060	0.115	0.283	0.113		0.072	0.111	0.114
IL-6	0.023	0.020	0.026	0.086	0.022		0.016	0.090	0.039
IL-8	0.172	0.072	0.356	0.285	0.270		0.102	0.207	0.321
IL-10	0.075	0.116	0.173	0.358	0.219		0.134	0.110	0.166
CD4-full	0.374	0.570	0.441		0.693		0.344	0.374	0.358
CD8a	0.853	0.547	1.275	2.144	1.741		0.697	0.993	0.940
CD8b	0.112	0.060	0.253	0.406	0.227		0.102	0.157	0.132
LGALS1	5.979	8.340	13.177	15.671	12.381		7.362	6.821	9.126
LGALS8	2.028	1.670	4.056	8.225	5.389		1.905	4.691	3.364
IgM	12.126	15.889	9.383		6.021		4.790	6.916	3.387
IgT	0.079	0.049	0.131		0.356		0.040	0.051	0.117
IgT-m			1.110	2.313	2.969			0.678	0.727
mIgM	0.180	0.209	0.293	1.050	0.646	0.420	0.166	0.312	0.174
SIRT1	0.637	0.514	0.933	1.133	0.841	1.945	0.547	0.651	0.818
SIRT2	0.732	0.966	1.079	1.569	1.248	1.840	0.674	1.223	0.779
SIRT3	0.275	0.454	0.441	0.801	0.521	0.986	0.283	0.435	0.463
SIRT4	0.093	0.070	0.139	0.203	0.141	0.224	0.084	0.110	0.125
SIRT5	1.057	1.141	1.404	1.404	1.079	1.803	0.940	1.087	1.214
SIRT6	0.144	0.167	0.241	0.403	0.247	0.409	0.195	0.160	0.264
SIRT7	0.154	0.182	0.287	0.351	0.255	0.603	0.124	0.144	0.225
